# Polyphenol‐Rich Duhuo Jisheng Decoction Enhances Mesenchymal Stem Cell–Derived Exosome–Mediated Chondroprotection via PI3K/AKT Signaling in Osteoarthritis

**DOI:** 10.1002/fsn3.71867

**Published:** 2026-05-27

**Authors:** Zhouwei Liao, Weiran Wang, Shunwan Jiang, Lixin Wang, Tian Yu, Xinqiang Ni, Shaoqun Zhang, Jun Liu, Junzheng Yang, Hui Xie

**Affiliations:** ^1^ Orthopaedics Department Shenzhen Traditional Chinese Medicine Hospital Shenzhen Guangdong China; ^2^ The Forth Clinical College of Guangzhou University of Chinese Medicine Shenzhen Guangdong China; ^3^ Guangdong Second Traditional Chinese Medicine Hospital (Guangdong Province Engineering Technology Research Institute of Traditional Chinese Medicine) Guangzhou Guangdong China; ^4^ The Research Team on Bone and Joint Degeneration and Injury Guangdong Provincial Academy of Chinese Medical Sciences Guangzhou Guangdong China; ^5^ The Fifth Clinical College of Guangzhou University of Chinese Medicine Guangzhou Guangdong China

**Keywords:** bone marrow mesenchymal stem cells, dietary polyphenols, Duhuo Jisheng Decoction, exosomes, non‐communicable chronic diseases, osteoarthritis, PI3K/AKT signaling

## Abstract

Osteoarthritis (OA), a representative chronic degenerative joint disease, is closely associated with chronic inflammation and dysregulated cell survival. Dietary polyphenols are recognized for their protective roles against chronic inflammatory conditions. Duhuo Jisheng Decoction (DHJST), a polyphenol‐rich traditional herbal formula, is widely used for degenerative joint diseases. This study aims to explore the novel mechanisms of DHJST‐derived bioactives in enhancing the chondroprotective potential of mesenchymal stem cell‐derived exosomes. We integrated network pharmacology and machine learning to identify the core AKT‐related targets of DHJST. Bone marrow mesenchymal stem cells (BMSCs) were preconditioned with DHJST‐containing serum to generate enhanced exosomes (DHJST‐Exos), applying to IL‐1β–stimulated rat chondrocytes and a collagenase‐induced OA rat model. The therapeutic efficacy was evaluated via inflammatory markers, extracellular matrix (ECM) homeostasis, and apoptosis assays, with the PI3K/AKT inhibitor Rigosertib used for mechanistic validation. Network pharmacology identified key bioactive polyphenols including wogonin, quercetin, kaempferol, and other polyphenol‐like constituents in DHJST that significantly modulate the PI3K/AKT pathway. DHJST ‐Exos effectively suppressed TNF‐α and IL‐6, reduced MMP‐13 and ADAMTS‐5 levels, and restored COL2A1 and Aggrecan expression. Furthermore, DHJST ‐Exos attenuated chondrocyte apoptosis by regulating the Caspase‐3/Bax/Bcl‐2 axis. Mechanistically, DHJST ‐Exos up‐regulated the expression of hub genes JUN and KDR via AKT phosphorylation, a protective effect that was significantly abrogated by Rigosertib. This study demonstrates that polyphenol‐rich DHJST enhances the therapeutic efficacy of BMSC‐derived exosomes against OA by activating the AKT signaling pathway and its key nodes, JUN and KDR. This synergistic strategy, combining dietary bioactives with exosome‐mediated delivery, represents a promising adjunctive approach for the management of chronic degenerative joint diseases.

## Graphical abstract

This graphical abstract illustrates the chondroprotective mechanism of exosomes derived from bone marrow mesenchymal stem cells (BMSCs) preconditioned with polyphenol‐rich Duhuo Jisheng Decoction (DHJST‐Exos). Enriched with bioactive compounds such as wogonin, quercetin, and kaempferol, DHJST‐Exos effectively ameliorate osteoarthritis (OA). Mechanistically, DHJST‐Exos activate the PI3K/AKT signaling pathway, subsequently upregulating the downstream targets JUN and KDR. This regulatory cascade leads to the suppression of inflammatory factors, restoration of extracellular matrix (ECM) homeostasis, and inhibition of chondrocyte apoptosis. The specific PI3K/AKT inhibitor Rigosertib effectively abrogates these protective effects, confirming the essential role of this pathway in DHJST‐Exos‐mediated OA therapy.

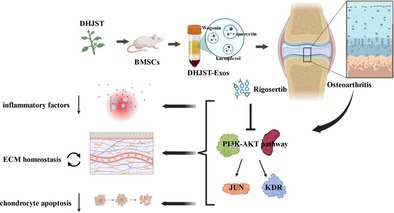

## 1 Introduction

Osteoarthritis (OA) is a prevalent chronic degenerative joint disease characterized by progressive articular cartilage destruction, subchondral bone remodeling, and synovial inflammation. It commonly affects the knee, hip, ankle, hand, and spine, leading to pain, stiffness, and reduced mobility. As a leading cause of disability worldwide, the incidence of OA increases with age, seriously affecting quality of life. Current clinical management primarily relies on surgical interventions such as joint replacement and cartilage repair, combined with basic treatments like rehabilitation exercise and education (Glyn‐Jones et al. 2015; Jiang 2022; Mandl 2019; Abramoff and Caldera 2020). Analgesics and targeted drugs serve as adjuvant therapies. Among them, bone marrow mesenchymal stem cells (BMSCs) and their exosomes are an emerging treatment strategy for OA (Giorgino et al. 2023; Jin et al. 2021).

Initially, BMSCs gained attention in orthopedics due to their multi‐differentiation ability (Huang et al. 2009), which allows them to fill bone cell defects (Salonius et al. 2020; Yu et al. 2022). However, current evidence suggests that BMSCs primarily stimulate cartilage regeneration and inhibit inflammation by secreting cytokines rather than acting as a simple cell substitute (Barry 2019). Recent studies confirmed that BMSCs secrete exosomes that play a pivotal role in OA treatment (Ni et al. 2020). These exosomes act as mediators of intercellular communication (Cosenza et al. 2017; El Andaloussi et al. 2013), regulating immune responses and inhibiting cell apoptosis to suppress OA progression (Vonk et al. 2018). Exploring the molecular mechanism of these exosomes and improving their therapeutic potential are current focal points in OA research.

Traditional Chinese medicine has shown a significant role in enhancing the function of BMSCs and their exosomes (Barbalho et al. 2019). DHJST, a traditional herbal formula that has shown favorable clinical efficacy in treating OA. DHJST contains key polyphenolic compounds such as quercetin, wogonin, and kaempferol, which are widely distributed in natural dietary sources like berries, green tea, and various medicinal herbs. Recent reviews have highlighted that these food‐derived bioactives exert potent anti‐inflammatory effects in chronic conditions like OA by modulating oxidative stress and cellular survival pathways (Yang et al. 2025; Yue et al. 2026). Modern pharmacological research indicates that DHJST exhibits significant anti‐inflammatory and chondroprotective effects both in vivo and in vitro (Zhang et al. 2016; Zhao et al. 2022; Chan et al. 2023; Shi et al. 2022). For BMSCs, DHJST also promotes differentiation and proliferation (Li et al. 2010). However, the evidence for the combined application of DHJST and BMSCs remains insufficient.

In the mechanism studies of DHJST for OA, its regulatory effect on the classic signal PI3K‐AKT signaling pathway has been observed (Xin et al. 2023). PI3K‐AKT is a key signal governing chondrocyte autophagy imbalance, apoptosis, and aging in OA (Sun et al. 2020). Abnormalities in this pathway are frequently detected in chondrocytes and rat models. AKT, as a major downstream effector of PI3K, regulates biological activities such as survival and proliferation (Sun et al. 2020; Xu et al. 2021). BMSCs‐derived exosomes have been shown to modulate PI3K‐AKT signaling to promote chondrocyte survival (Wang et al. 2021; Zhang et al. 2019). Whether DHJST enhances the PI3K‐AKT signaling regulation of BMSCs on chondrocytes remains to be elucidated.

The purpose of this study was to determine whether pre‐treatment of BMSCs with DHJST‐containing serum could improve the therapeutic potential of their secreted exosomes in OA models. We isolated exosomes from BMSCs incubated with DHJST‐containing serum and evaluated their effects on IL‐1β‐induced OA chondrocytes and a rat OA model. By examining the PI3K–AKT signaling pathway, these results aim to provide experimental evidence for a potential strategy combining dietary bioactives with BMSC‐derived exosomes for OA treatment.

## 2 Methods

### 2.1 Screening of DHJST Bioactive Compounds and Target Prediction

The active components of the 15 herbs in DHJST are retrieved from the TCM Systems Pharmacology Database and Analysis Platform (TCMSP). Molecules are screened based on oral bioavailability (OB, %) ≥ 30% and drug‐like properties (DL) ≥ 0.18. Potential binding targets for these polyphenolic and polyphenol‐like small molecules are subsequently obtained. The herbal medicine‐active ingredient‐target network is constructed and visualized using Cytoscape v3.9.1.

### 2.2 Network Pharmacology and Enrichment Analysis

The Molecular Complex Detection (MCODE) module is applied to identify core network clusters within the target interaction network. The Maximum Correntropy Criterion (MCC) algorithm of the Cytohubba plug‐in is utilized to calculate core nodes. Functional enrichment analysis, including Gene Ontology (GO) and Kyoto Encyclopedia of Genes and Genomes (KEGG) pathways, is performed using “ClusterProfiler” v4.6.2 to identify key signaling pathways modulated by DHJST.

### 2.3 Osteoarthritis Dataset Acquisition and Differential Expression Analysis

Four transcriptome sequencing (RNA‐seq) datasets (GSE111357, GSE107308, GSE207881, and GSE168505) are retrieved from the Gene Expression Omnibus (GEO) database. After batch effect removal using the “edgeR” package, the “limma” package is employed to identify significantly differentially expressed genes (DEGs) in OA cartilage tissue with thresholds of |log_2_ Fold Change| > 1 and adj.*p* < 0.05. The details of the datasets are summarized in Table S2.

### 2.4 Gene Set Variation Analysis (GSVA)

To quantify the functional status of the AKT signaling and cell death‐related pathways in individual samples, Gene Set Variation Analysis (GSVA) is performed using the “GSVA” v3.16.0 package. The expression matrix subsets, comprising the 71 key DHJST‐targeted genes within the AKT pathway (identified from core cluster 1), are extracted from the GSE111357 (merged with GSE107308), GSE207881, and GSE168505 datasets. Variation scores for each sample are calculated for the AKT and cell death‐related gene sets. Subsequently, the “limma” package is employed to identify significant differences in these pathway scores between OA and control groups, providing a quantitative basis for the involvement of AKT‐mediated signaling in OA pathogenesis.

### 2.5 Identification of AKT‐Related Hub Genes via Machine Learning

To identify critical genetic signatures, two unsupervised learning methods are integrated using the expression matrices of the 71 DHJST‐targeted genes in the AKT pathway. In Lasso Regression, the “glmnet” package is used with 10‐fold cross‐validation to determine the optimal regularization coefficient for feature selection. In Support Vector Machine (SVM), the “e1071” package is used to screen characteristic genes. The intersection of these models defines the final 11 hub genes, and their predictive accuracy is evaluated using area under the curve (AUC) analysis via the “pROC” package.

### 2.6 Molecular Docking Validation

The 3D structures of identified hub proteins such as JUN and KDR are retrieved from the RCSB‐PDB database. Molecular docking is conducted using PyMOL and AutoDock Vina to calculate binding affinities between DHJST active ingredients and core target residues. Binding sites with a distance less than 3 Å are selected for visualization.

### 2.7 Preparation of Duhuo Jisheng Decoction

The preparation of DHJST was performed by The Chinese Hospital. The decoction consists of 15 herbal components: Angelicae Pubescentis Radix, Taxilli Herba, Eucommiae Cortex, Achyranthes Bidentatae Radix, Asari Radix cum Rhizoma, Gentianae Macrophyllae Radix, Poria, Cinnamomi Cortex, Saposhnikoviae Radix, Ligustici Chuanxiong Rhizoma, Angelicae Sinensis Radix, Paeoniae Radix Alba, Rehmanniae Radix Praeparata, Ginseng Radix et Rhizoma, and Glycyrrhizae Radix et Rhizoma. The detailed composition, including the weight and source of each herb, is summarized in Table S1. In brief, the mixture was boiled in 1000 mL of distilled water until the liquid was concentrated to 500 mL. The residue was then filtered and collected for subsequent experiments. The herbal names and quality standards strictly follow the Pharmacopeia of the People's Republic of China.

### 2.8 Preparation of Drug‐Containing Serum

Male Sprague–Dawley (SD) rats in Specific Pathogen Free grade, weighing 180–220 g and aged 8 weeks, were purchased from the Guangdong Experimental Animal Center and housed in the animal facility. The research was conducted in accordance with ethical standards and received approval from the Animal Ethics Committee of Guangzhou University of Chinese Medicine (Approval No.: 20210304032). Rats (*n* = 10) were randomly assigned using a random number table, with body weights balanced among groups. Rats were administered DHJST stock solution via gavage twice daily for 5 consecutive days. On the 5th day, 1.5 h after the final dose, rats were anesthetized with 2% isoflurane. Blood was collected from the abdominal aorta, cooled for 30 min, and centrifuged at 3500 rpm for 3 min at 25°C. The collected serum was inactivated in a 56°C water bath for 30 min. Finally, euthanasia was performed via cervical dislocation.

### 2.9 BMSC Culture and DHJST‐Exos Preparation

Primary rat BMSCs were isolated from the femur and tibia and cultured in low‐glucose Dulbecco's modified Eagle medium (DMEM) supplemented with 10% Fetal Bovine Serum (FBS) and 1% penicillin–streptomycin under conditions of 5% CO_2_ and 37°C. The medium was refreshed every 3 days. Third‐generation BMSCs at 70%–80% confluence were incubated for 48 h with complete medium containing 10% drug‐containing serum, filtered with a 0.22 μM filter, to obtain DHJST‐BMSCs‐Exomes.

### 2.10 Exosome Isolation and Characterization

Exosomes were isolated via differential ultracentrifugation: 1000 g (10 min), 2000 g (10 min), 10,000 g (30 min), and finally 100,000 g for 70 min. The precipitate was washed in PBS and centrifuged again at 100,000 g for 70 min. For TEM observation, exosomes were fixed in dimethoate buffer (3% glutaraldehyde and 2% paraformaldehyde), dispensed onto copper grids, stained with 2% uranyl acetate, and observed at 120 kV. For Nanoparticle Tracking Analysis (NTA), the concentration and particle size distribution of the isolated exosomes were precisely quantified using NTA. Samples were diluted with PBS to an appropriate concentration and analyzed to determine the peak particle diameter and particle density. For Flow Cytometry, 50 μL exosomes were incubated with magnetic beads, blocked with casein buffer following the Beijing Novo Bio kit protocol, and labeled with CD9, CD63, and CD81 antibodies. Levels were quantified using the staining index (SI).

### 2.11 Primary Chondrocyte Culture and Treatment

Cartilage tissues from the knee joints of SD rats were digested overnight with 0.2% type II collagenase. Cells were cultured in DMEM‐F12 medium with 10% FBS and 25 μg/mL L‐ascorbic acid 2‐phosphate. Second‐generation chondrocytes were stimulated with 1 ng/mL IL‐1β for 24 h to establish the OA model. Subsequently, DHJST‐BMSCs‐Exomes or BMSCs‐Exomes were applied for 24 h.

### 2.12 Osteoarthritis Rat Model Construction

Twenty male SD rats were randomly assigned (*n* = 5 per group) to Control, Model, DHJST, and DHJST_Rigosertib groups using a random number table. OA model was induced by intra‐articular injection of 100 μL of type II collagenase (2 mg/mL) on day 0 and day 7. The DHJST group received 100 μL DHJST‐BMSCs‐Exomes (10^6^ particles mL^−1^ day^−1^), while the DHJST_Rigosertib treatment group received 200 mg kg^−1^ day^−1^ Rigosertib. At the end of the experimental period (day 14), the rats were euthanized, and samples of peripheral blood, synovial fluid, and knee cartilage tissue were procured for further analysis.

### 2.13 ELISA, Western Blot, and qPCR

For all molecular assays, three independent biological replicates were performed. TNF‐α, IL‐6, MMP‐13, ADAMTS‐5, COL2A1, and Aggrecan levels were measured at 492 nm for ELISA assay. In Western blot, cartilage tissue and chondrocytes were lysed in RIPA + cocktail buffer. 20 μg of protein was separated by 10% SDS‐PAGE gel, transferred to PVDF membranes, and incubated with primary antibodies (KDR, JUN, PI3K, t‐AKT, p‐AKT, Caspase3, Bax, Bcl2). GAPDH served as the internal control. In RT‐qPCR, RNA was extracted via Trizol and reverse‐transcribed. Target gene expression relative to GAPDH was calculated using the 2^−ΔΔ*Ct*^ method.

### 2.14 Statistical Methods

Bioinformatics analysis was performed in R v4.2.2 language and its visualization software Rstudio v2022.12.0 + 353 (2022.12.0 + 353). Experimental data were statistically analyzed using GraphPad Prism 10. The statistics were all in line with the normal distribution and expressed as mean ± SD. The differences between groups were tested by Student's *t*‐test. *p* value less than 0.05 indicated that the difference between the groups was statistically significant.

## 3 Results

### 3.1 Network Pharmacology Identifies PI3K‐AKT as the Primary Target Pathway of DHJSD

**FIGURE 1:**
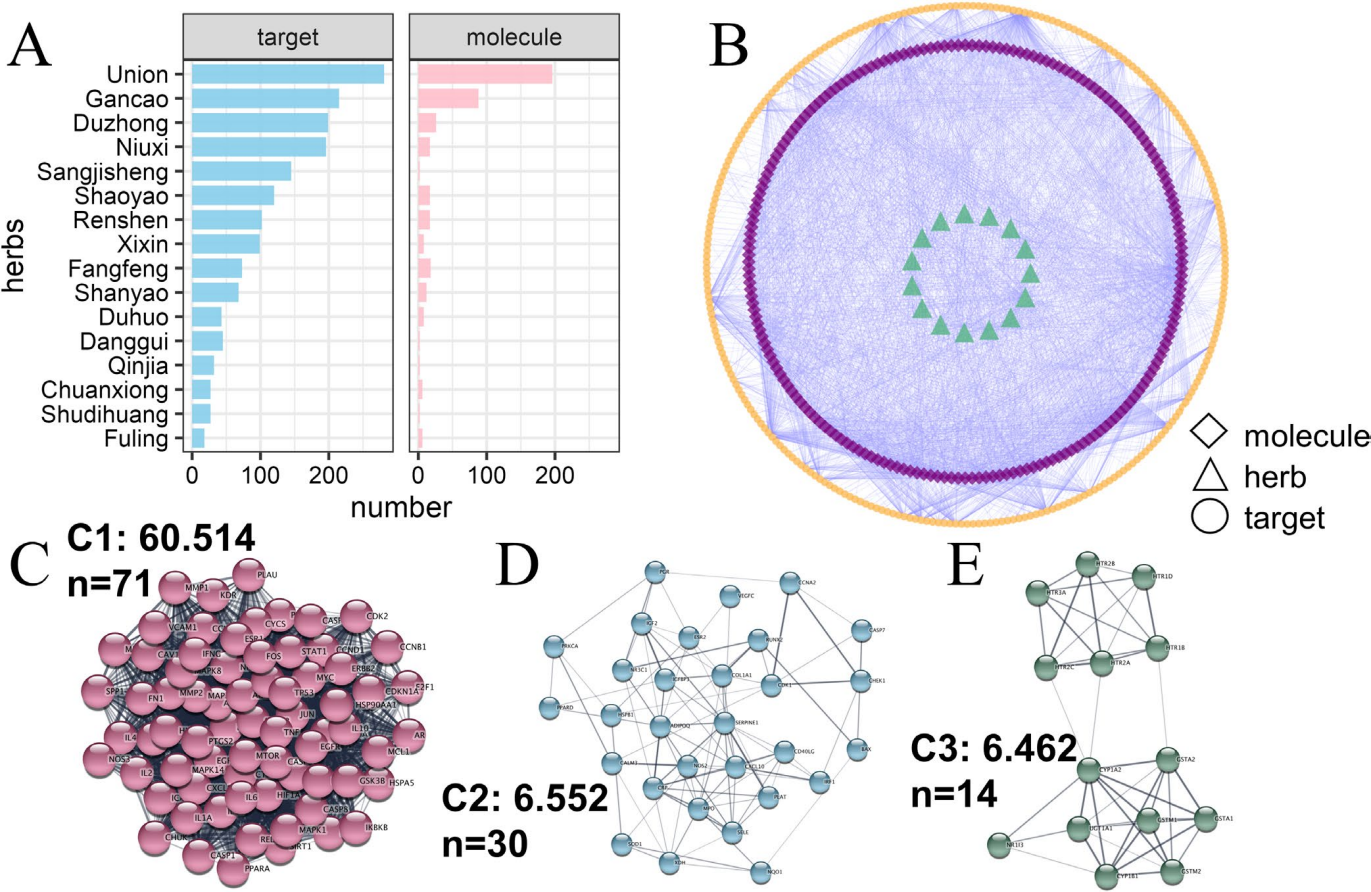
Network pharmacology analysis of active ingredients and targets in DHJST. (A) Distribution of active ingredients and their corresponding targets across the 15 constituent herbs, with *Gancao*, *Duhuo*, and *Niuxi* possessing the highest constituent counts; (B) Herbal medicine–active ingredient–target network visualization; (C–E) Identification of the top three core network clusters (Cluster 1, 2, and 3) housing 71, 30, and 14 genes, respectively, representing the pivotal functional modules of DHJST.

In this study, 196 active components of DHJST and 281 corresponding target genes were obtained from the TCMSP database. The intricate interactions between these 15 herbal medicines, their bioactive molecules, and target genes are visualized in the network diagram (Figure 1A,B). Utilizing the Molecular Complex Detection (MCODE) method, three core network clusters (*k* > 6) are identified within the target interaction network (Figure 1C–E).

**FIGURE 2:**
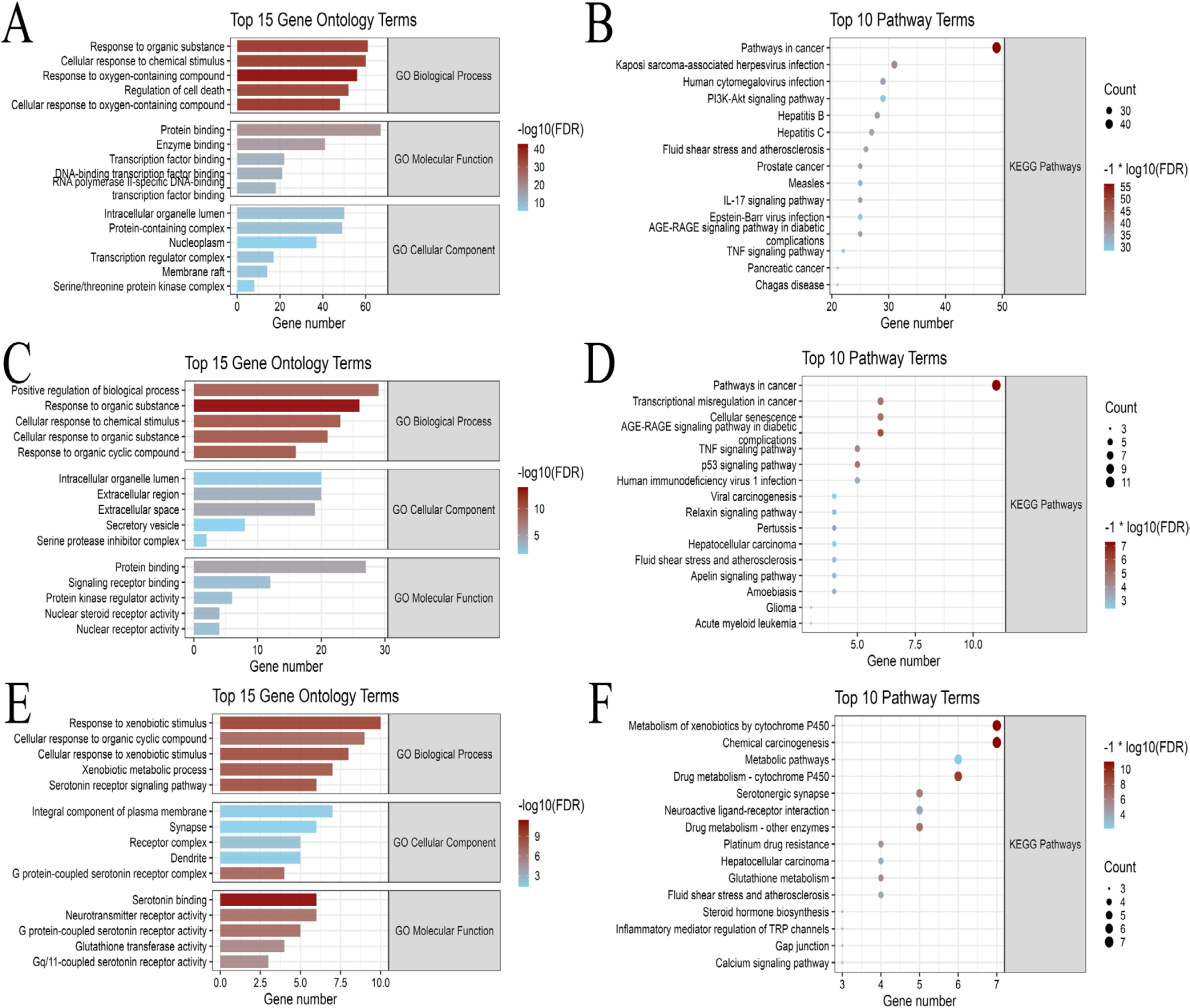
Functional enrichment of Core DHJST target networks. (A, B) Gene Ontology (GO) and KEGG pathway enrichment analysis for core cluster 1, highlighting the dominance of the PI3K‐AKT signaling pathway; (C, D) Functional enrichment profiles for core cluster 2; (E, F) GO and KEGG enrichment results for core cluster 3.

Functional enrichment analysis reveals that core cluster 1 (71 genes) is significantly enriched in biological processes (BP) related to chemical stimulus response and the regulation of cell death. Notably, Kyoto Encyclopedia of Genes and Genomes (KEGG) analysis indicates that these targets are predominantly involved in the PI3K‐AKT signaling pathway, alongside immune‐related pathways such as IL‐17 and TNF signaling (Figure 2A,B). Sub‐level core networks (C2 and C3) are primarily associated with xenobiotic metabolism and additional immune regulation pathways (Figure 2C–F). These results collectively suggest that the therapeutic efficacy of DHJST bioactives is fundamentally mediated through the modulation of the PI3K‐AKT signaling axis and cellular survival mechanisms.

### 3.2 DEGs in OA Cartilage Are Primarily Enriched in the PI3K‐AKT and Immune‐Related Pathways

**FIGURE 3:**
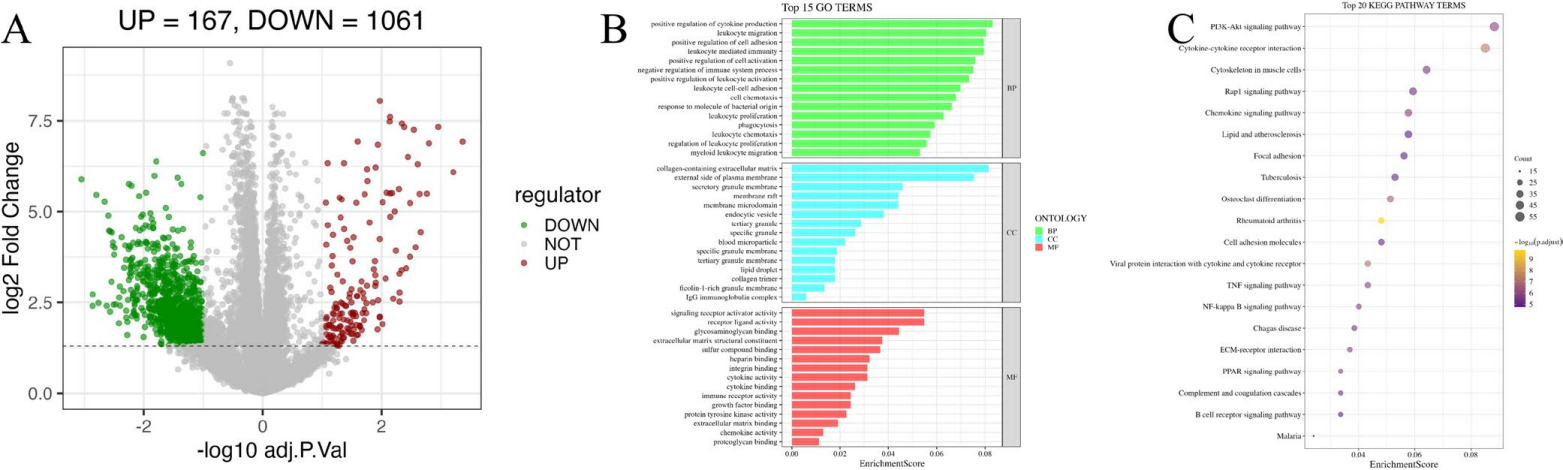
Transcriptomic landscape and functional enrichment in OA cartilage tissue. (A) Volcano plot of DEGs, showing 1061 down‐regulated and 167 up‐regulated genes in OA compared to controls; (B) Top 15 enriched GO terms across biological processes (BP), cellular components (CC), and molecular functions (MF); (C) Top 20 enriched KEGG pathways, highlighting the dominance of the PI3K‐AKT signaling pathway.

Differential expression analysis of OA cartilage tissue identifies 1228 DEGs, comprising 1061 down‐regulated and 167 up‐regulated genes (Figure 3A). Functional enrichment analysis reveals that these DEGs are significantly involved in the PI3K‐AKT signaling pathway (Figure 3C), aligning with the target pathways of DHJST.

Across three independent OA datasets (GSE111357, GSE207881, and GSE168505), GSVA scores indicate consistent dysregulation of the AKT axis. Specifically, AKT1‐mediated signaling is significantly diminished in OA samples, whereas scores for cell death, mitophagy, and autophagic death‐related pathways are notably elevated (Figure S1A–C). These findings suggest that the suppression of AKT1 signaling and the activation of programmed cell death pathways exert opposing regulatory effects during OA progression.

Furthermore, Gene Ontology (GO) analysis delineates that these DEGs are profoundly enriched in immune‐related biological processes, including cytokine production regulation, leukocyte migration, and leukocyte‐mediated immunity (Figure 3B). Significant enrichment is also observed in cellular components such as the collagen‐containing extracellular matrix and endocytic vesicles, as well as molecular functions like receptor ligand and cytokine activity (Figure 3B). Collectively, the aberrant expression of these immune and matrix‐related genes highlights the critical role of inflammatory initiation and AKT signaling impairment in OA cartilage destruction.

### 3.3 Identification of 11 Hub Genes Associated With AKT Signaling via Machine Learning

To pinpoint the most critical genetic signatures targeted by DHJSD within the AKT axis, unsupervised machine learning is applied to the 71 candidate genes. Lasso regression identifies 10 significant feature genes such as IL6, KDR, and CASP8 with a minimum lambda value of −9.015 and prediction accuracies exceeding 0.9. SVM methodology screens two characteristic genes, CAV1 and JUN, which also exhibit high diagnostic performance (AUC > 0.9).

To further validate the clinical relevance of these targets, a Pearson correlation and overlap analysis were performed. The results showed a significant intersection among DHJST targets, AKT signaling genes, and OA‐specific DEGs, with 17 genes overlapping across all three sets (Figure S3A). Furthermore, a correlation matrix confirmed that core hub genes such as *JUN* exhibit a moderate positive correlation with *AKT1* expression in OA cartilage (Figure S3B).

**FIGURE 4:**
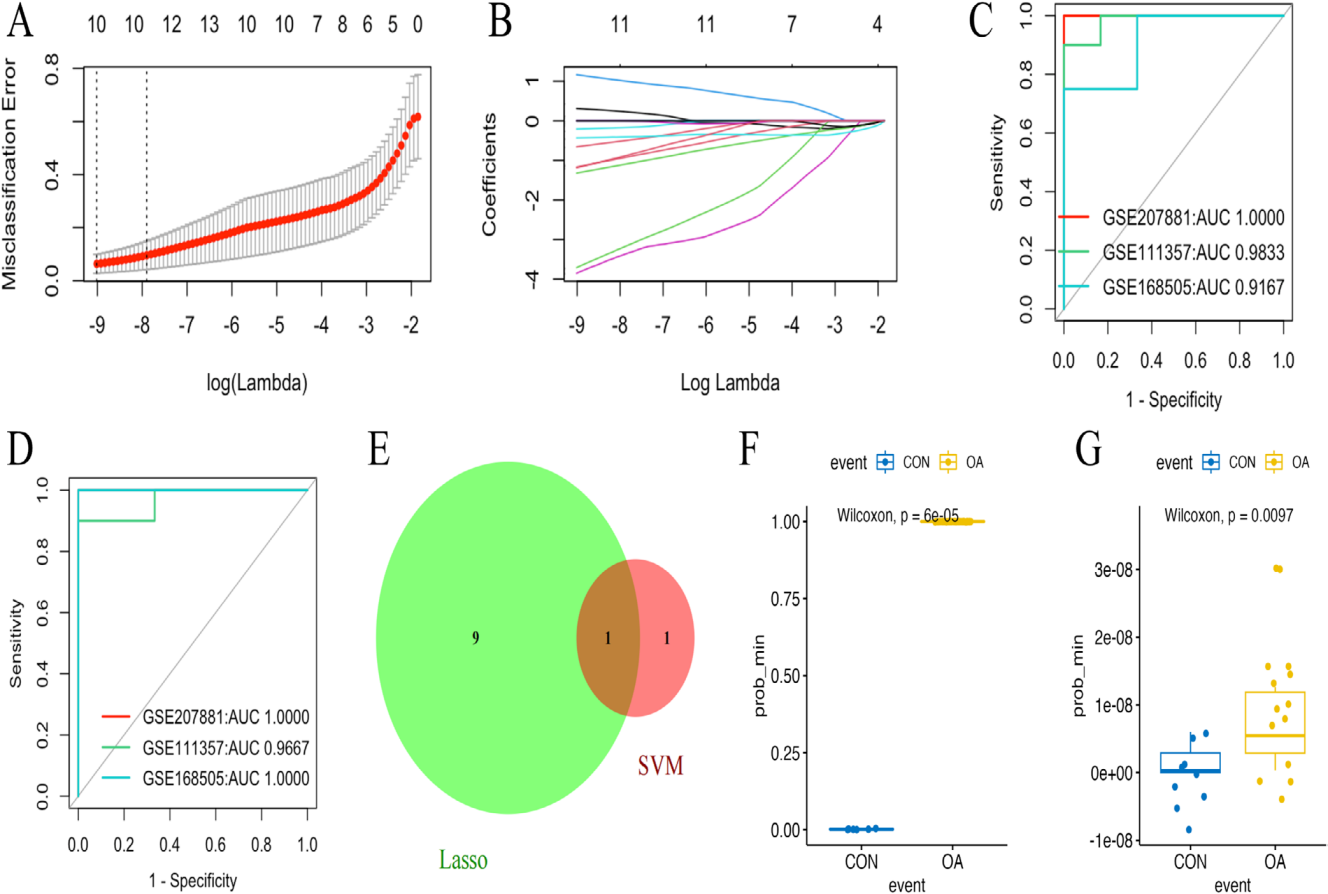
Screening of OA characteristic genes using integrated machine learning. (A–C) Lasso regression analysis for feature gene identification and cross‐validation; (D) SVM model prediction accuracy; (E) Venn diagram showing the overlap of characteristic genes; (F, G) Predictive scores of the 11 gene combined model in training and validation sets.

**FIGURE 5:**
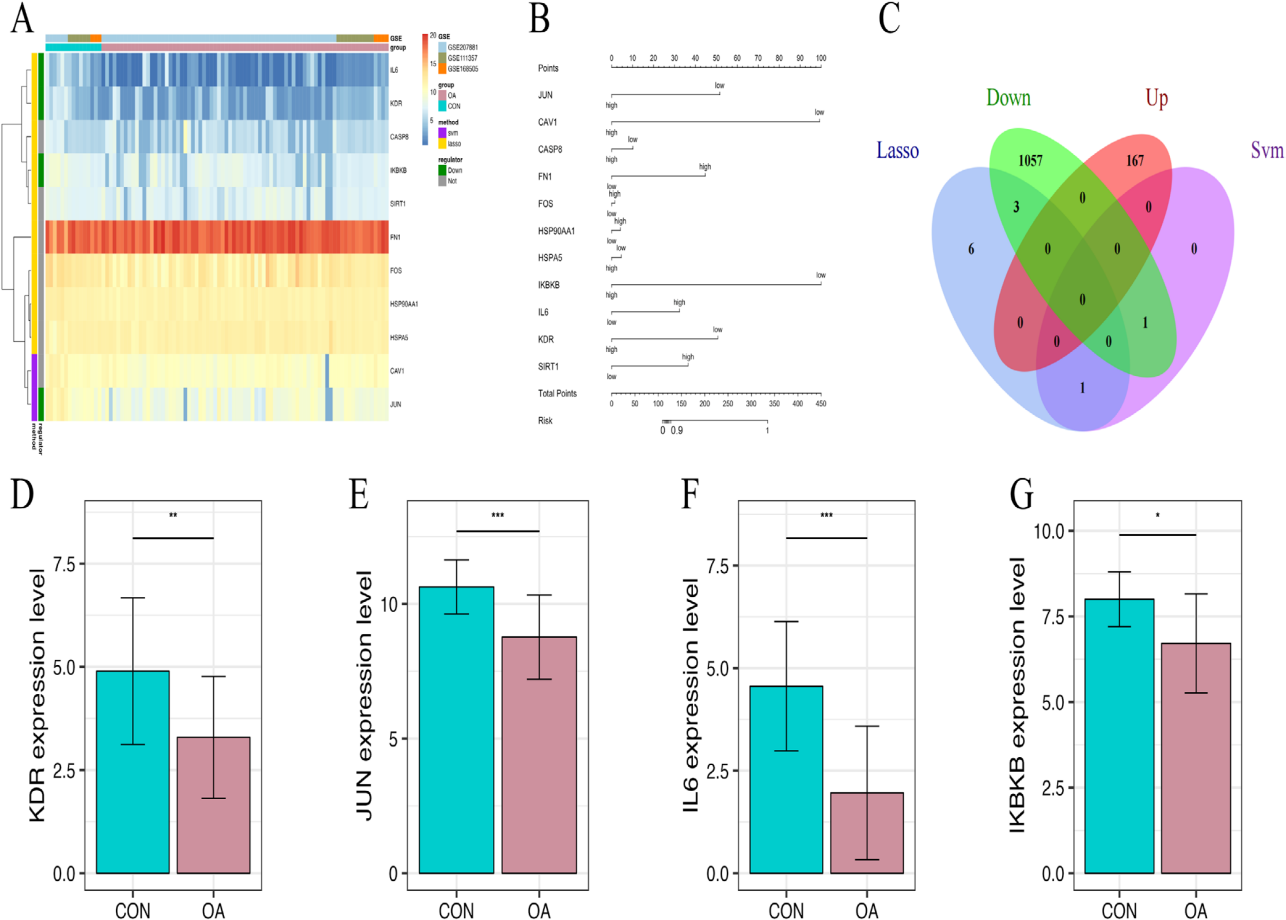
Expression profiles and contribution analysis of the 11 hub genes. (A) Heatmap of the 11 hub genes across OA and control samples; (B) Logistic regression evaluating the contribution of each gene to OA prediction; (C–G) Quantitative comparison of expression levels for core genes *KDR*, *JUN*, *IL6*, and *IKBKB*. The difference between the two groups is statistically significant. *P < 0.05, **P < 0.01, ***P < 0.001.

The intersection of these models results in 11 hub genes, which effectively distinguish OA samples from controls in both training and validation sets (Figure 4F–G). Analysis of contribution scores reveals that the down‐regulation of KDR, JUN, and IKBKB serves as a primary driver of OA pathogenesis (Figure 5B–G). After screening for drug‐likeness and bioavailability, nine representative bioactive polyphenols (e.g., quercetin, wogonin, and kaempferol) that passed all filters were identified for further analysis (Table 1).

**TABLE 1:** Relationship between Duhuo Jisheng Decoction and 4 core targets related to AKT signal.

Mol ID	Molecule name	OB (%)	DL	Herb	Targets	PubChem ID	Drug‐likeness rules
MOL000098	Quercetin	46.43	0.28	Duzhong, Sangjisheng, Gancao, Niuxi	IL6, JUN	5280343	Passed
MOL000173	Wogonin	30.68	0.23	Fangfeng, Niuxi	IL6, JUN, KDR	5281703	Passed
MOL000422	Kaempferol	41.88	0.24	Duzhong, Gancao, Niuxi, Renshen, Xixin, Shaoyao	JUN, IKBKB	5280863	Passed
MOL001460	Cryptopin	78.74	0.72	Xixin	KDR	72616	Passed
MOL002135	Myricanone	40.6	0.51	Chuanxiong	KDR	161748	Passed
MOL004824	(2S)‐6‐(2,4‐dihydroxyphenyl)‐2‐(2‐hydroxypropan‐2‐yl)‐4‐methoxy‐2,3‐dihydrofuro[3,2‐g]chromen‐7‐one	60.25	0.63	Gancao	KDR	637112	Passed
MOL004904	Licopyranocoumarin	80.36	0.65	Gancao	KDR	122851	Passed
MOL004935	Sigmoidin‐B	34.88	0.41	Gancao	KDR	73205	Passed
MOL009055	hirsutin_qt	49.81	0.37	Duzhong	KDR	9794659	Passed

*Note:* OB, oral availability (%), screening condition is > 30%; DL, drug‐like property, screening condition is > 0.18. Nine small molecules that are predicted to be in line with drug principles by SWISS‐ADMET and screened out from 26 active ingredients. Drug‐likeness rules include Lipinski, Pfizer, GSK, and Golden Triangle criteria. All nine compounds passed these filters.

### 3.4 Molecular Docking Confirms Strong Affinities Between DHJSD Bioactives and Core Hub Proteins

**FIGURE 6:**
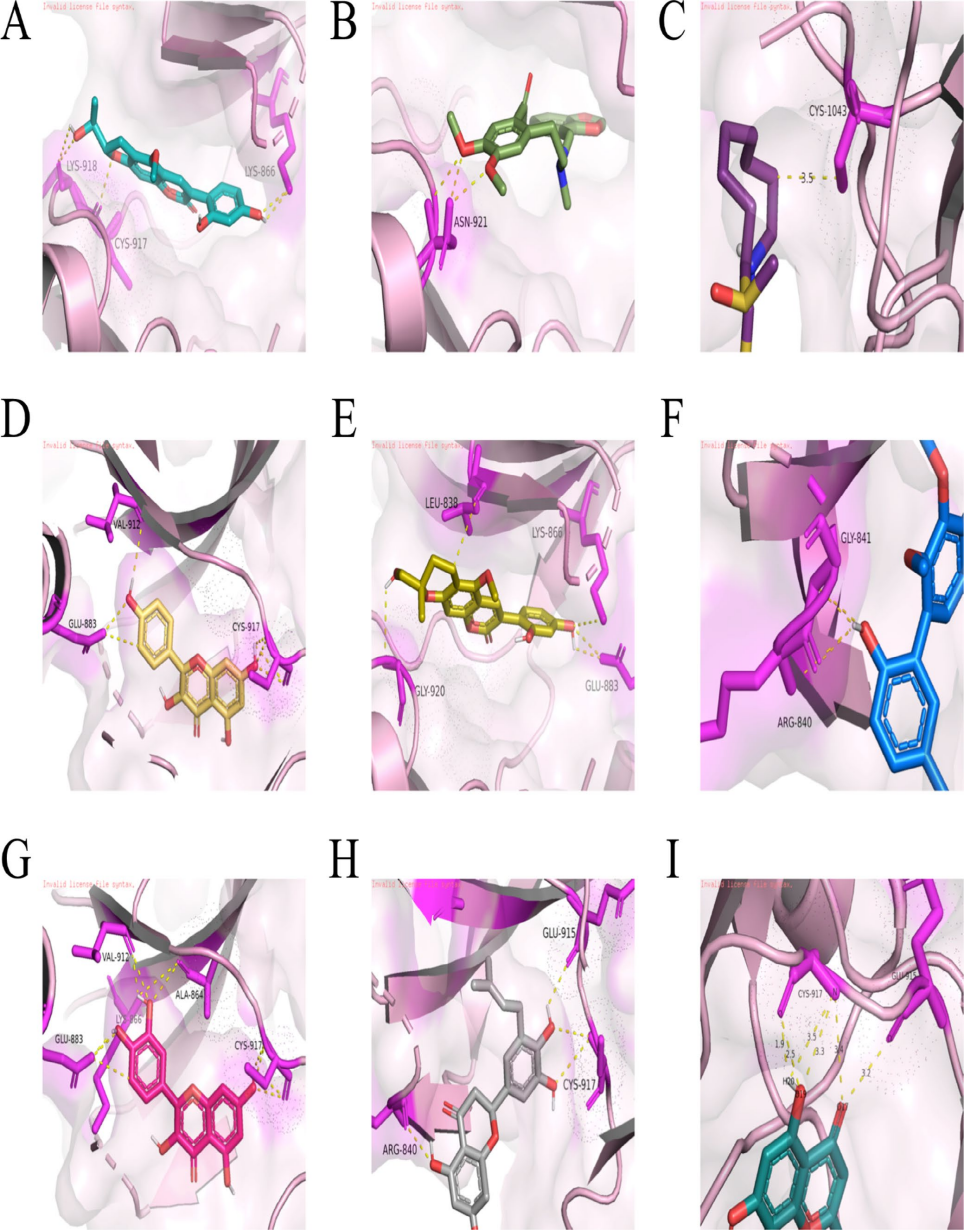
Molecular docking validation of core polyphenolic bioactives with KDR. (A–I) Visualizations of the stable binding conformations and detailed molecular interactions between 9 effective small molecules and the active pocket of the KDR protein (PDB: 1y6b; 2‐764 aa): Wogonin (A), quercetin (B), kaempferol (C), hirsutin_qt (D), Sigmoidin‐B (E), Myricanone (F), licopyranocoumarin (G), Cryptopin (H), and (2S)‐6‐(2,4‐dihydroxyphenyl)‐2‐(2‐hydroxypropan‐2‐yl)‐4‐methoxy‐2,3‐dihydrofuro[3,2‐g]chromen−7‐one (I). The key interaction networks highlight crucial hydrogen bonding and hydrophobic residues (e.g., CYS‐917, GLU‐915). All core compounds exhibit high binding affinities (below −7.0 kcal/mol), supporting their potential role as primary activators of the AKT signaling axis in DHJST.

**FIGURE 7:**
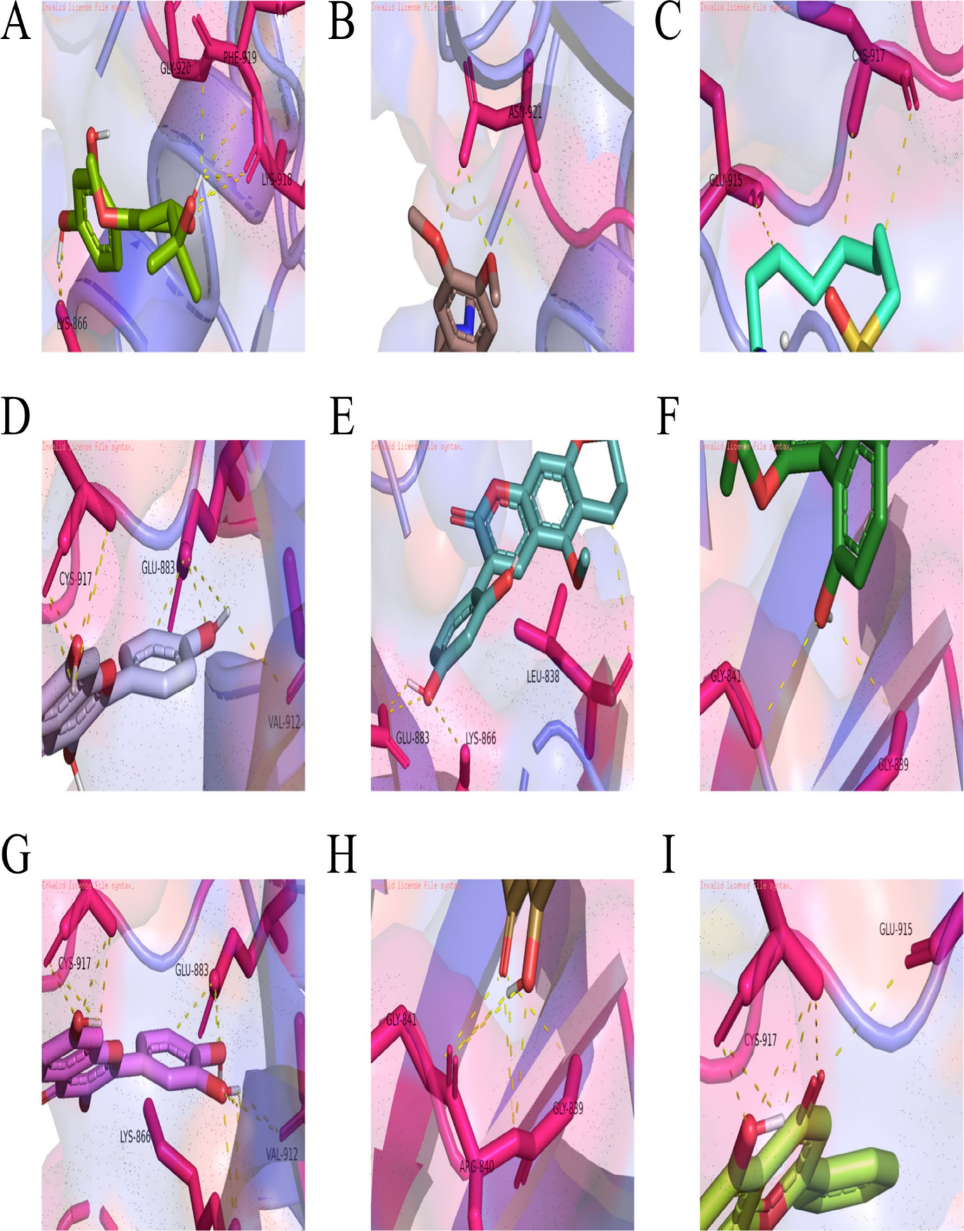
Molecular docking validation of core polyphenolic bioactives with the catalytic domain of KDR (PDB: 3v2a, 806‐1171aa). (A–I) Structural models and detailed interaction analysis of 9 effective small molecules docking into the active site of the KDR protein: Wogonin (A), quercetin (B), kaempferol (C), hirsutin_qt (D), Sigmoidin‐B (E), Myricanone (F), licopyranocoumarin (G), Cryptopin (H), and (2S)‐6‐(2,4‐dihydroxyphenyl)‐2‐(2‐hydroxypropan‐2‐yl)‐4‐methoxy‐2,3‐dihydrofuro[3,2‐g]chromen‐7‐one (I). Key binding sites (e.g., GLU‐19, ARG‐28) are identified within the interaction networks. The low binding energy scores suggest that these dietary polyphenols can effectively stabilize the KDR protein structure, thereby modulating downstream signaling pathways in vascular or relevant target cells.

**FIGURE 8:**
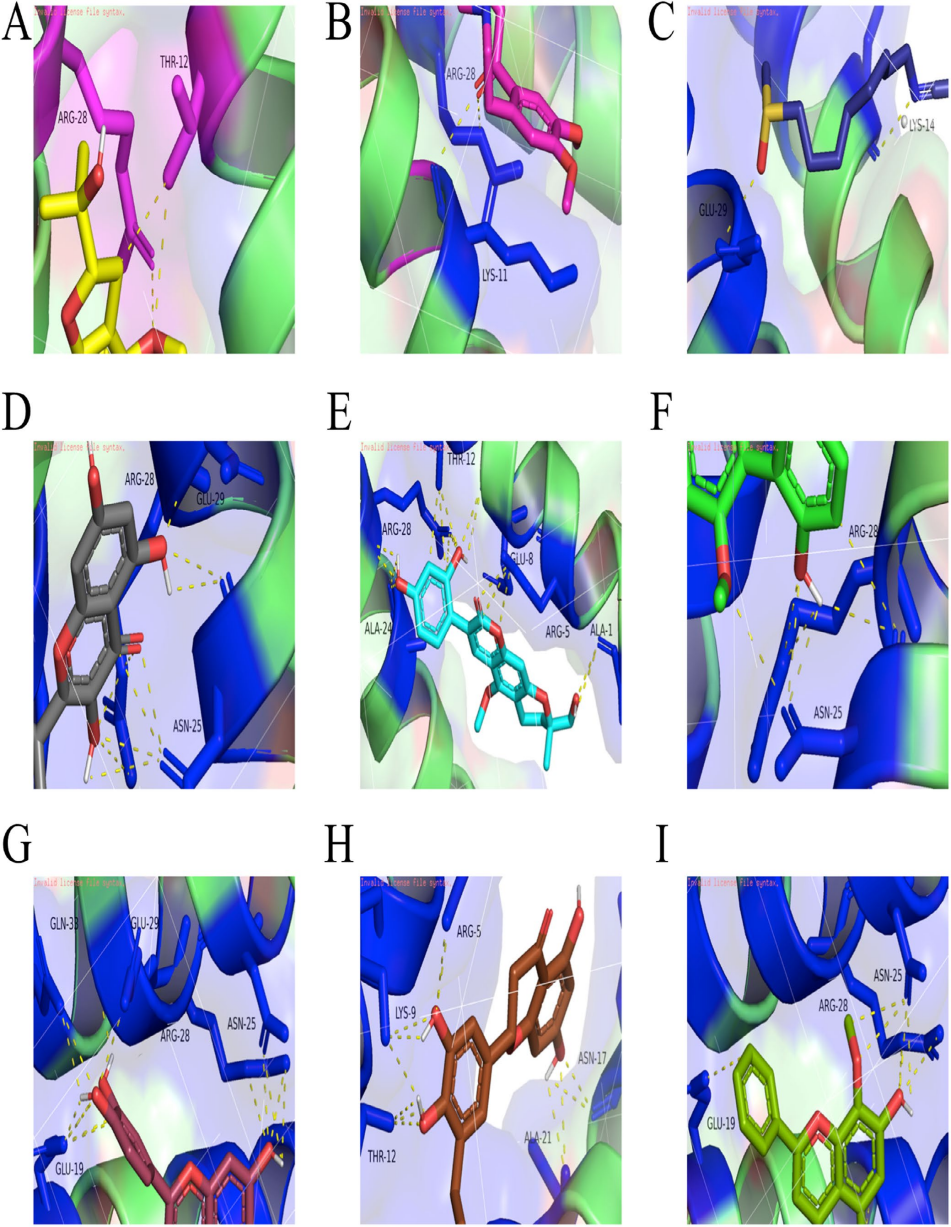
Comparative molecular docking analysis of DHJSD‐derived active molecules with c‐Jun (PDB: 5fv8). (A–I) Detailed view of the molecular interactions and binding configurations of 9 effective small molecules within the active domain of the JUN protein: Wogonin (A), quercetin (B), kaempferol (C), hirsutin_qt (D), Sigmoidin‐B (E), Myricanone (F), licopyranocoumarin (G), Cryptopin (H), and (2S)‐6‐(2,4‐dihydroxyphenyl)‐2‐(2‐hydroxypropan‐2‐yl)‐4‐methoxy‐2,3‐dihydrofuro[3,2‐g]chromen‐7‐one (I). Although all characteristic polyphenolic and polyphenol‐like constituents exhibit favorable binding scores, the primary therapeutic efficacy is predicted to be driven by the synergistic action of the core network‐hub polyphenols (quercetin, wogonin, and kaempferol) identified through the screening models.

Based on the machine learning results, JUN and KDR are identified as the most critical targets for DHJSD intervention. Network analysis reveals that 26 active ingredients of DHJSD directly target these four core nodes, with kaempferol notably targeting both *JUN* and *IKBKB* (Figure S2). Molecular docking results demonstrate that nine representative polyphenols, including quercetin, wogonin, and kaempferol, exhibit strong binding affinities (below −7 kcal/mol) with KDR (residues CYS‐917, GLU‐915) and JUN (residues GLU‐19, ARG‐28) (Figures 6, 7, 8, Table 2). In contrast, these small molecules show relatively weak affinity for IKBKB and IL6 (Figure S4). These findings suggest that DHJSD exerts its protective effects by directly binding to and potentially activating the KDR–AKT–JUN signaling axis.

**TABLE 2:** Affinity of the nine active ingredients of Duhuo Jisheng Decoction with two core target proteins.

PDB	Protein	Molecule	Affinity (kcal/mol)	rmsd.l.b.	rmsd.u.b.	Amino acid residues
1y6b	KDR	Wogonin	−8.5	0	0	CYS‐917, GLU‐915
1y6b	KDR	Quercetin	−8.7	0	0	ALA‐864, LYS‐866, GLU‐883, VAL‐912, CYS‐917
1y6b	KDR	Kaempferol	−8.2	0	0	CYS‐1043
1y6b	KDR	hirsutin‐qt	−3.7	0	0	GLU‐883, VAL‐912, CYS‐917
1y6b	KDR	SigmoidinB	−8.6	0	0	ARG‐840, GLU‐915, CYS‐917
1y6b	KDR	Myricanone	−7.8	0	0	ARG‐840, GLY‐841
1y6b	KDR	Licopyranocoumarin	−8.3	0	0	LEU‐838, LYS‐866, GLU‐883, GLY‐920
1y6b	KDR	Cryptopin	−8.2	0	0	ASN‐921
1y6b	KDR	CID637112	−8.3	0	0	LYS‐866, CYS‐917, LYS‐918
3v2a	KDR	Wogonin	−8.5	0	0	CYS‐917, GLU‐915
3v2a	KDR	Quercetin	−8.7	0	0	LYS‐866, GLU‐883, VAL‐912, CYS‐917
3v2a	KDR	Kaempferol	−8.2	0	0	GLU‐883, VAL‐912, CYS‐917
3v2a	KDR	hirsutin‐qt	−4.2	0	0	GLU‐915, CYS‐917
3v2a	KDR	SigmoidinB	−8.6	0	0	GLY‐839, ARG‐840, GLY‐841
3v2a	KDR	Myricanone	−7.8	0	0	GLY‐839, GLY‐841
3v2a	KDR	Licopyranocoumarin	−8.4	0	0	LEU‐838, LYS‐866, GLU‐883
3v2a	KDR	Cryptopin	−8.2	0	0	ASN‐921
3v2a	KDR	CID637112	−8.3	0	0	LYS‐866, LYS‐918, PHE‐919, GLY‐920
5fv8	cJUN	Wogonin	−6.3	0	0	GLU‐19, ASN‐25, ARG‐28
5fv8	cJUN	Quercetin	−6.4	0	0	GLU‐19, ASN‐25, ARG‐28, GLU‐29, GLN‐33
5fv8	cJUN	Kaempferol	−6.4	0	0	ASN‐25, ARG‐28, GLU‐29
5fv8	cJUN	hirsutin‐qt	−3.7	0	0	LYS‐14, GLU‐29
5fv8	cJUN	SigmoidinB	−7.6	0	0	ARG‐5, LYS‐9, THR‐12, ASN‐17, ALA‐21
5fv8	cJUN	Myricanone	−6.5	0	0	ASN‐25, ARG‐28
5fv8	cJUN	Licopyranocoumarin	−7	0	0	ALA‐1, ARG‐5, GLU‐8, THR‐12, ALA‐24, ARG‐28
5fv8	cJUN	Cryptopin	−7	0	0	LYS‐11, ARG‐28
5fv8	cJUN	CID637112	−7.2	0	0	THR‐12, ARG‐28

Molecular docking simulations confirmed that these polyphenolic bioactives exhibited high binding affinities for KDR and JUN proteins, with specific interactions at key amino acid residues (Table 2). PDB, Protein Data Bank database.

### 3.5 DHJSD‐Pretreated BMSC Exosomes Inhibit Chondrocyte Inflammation and Apoptosis In Vitro

**FIGURE 9:**
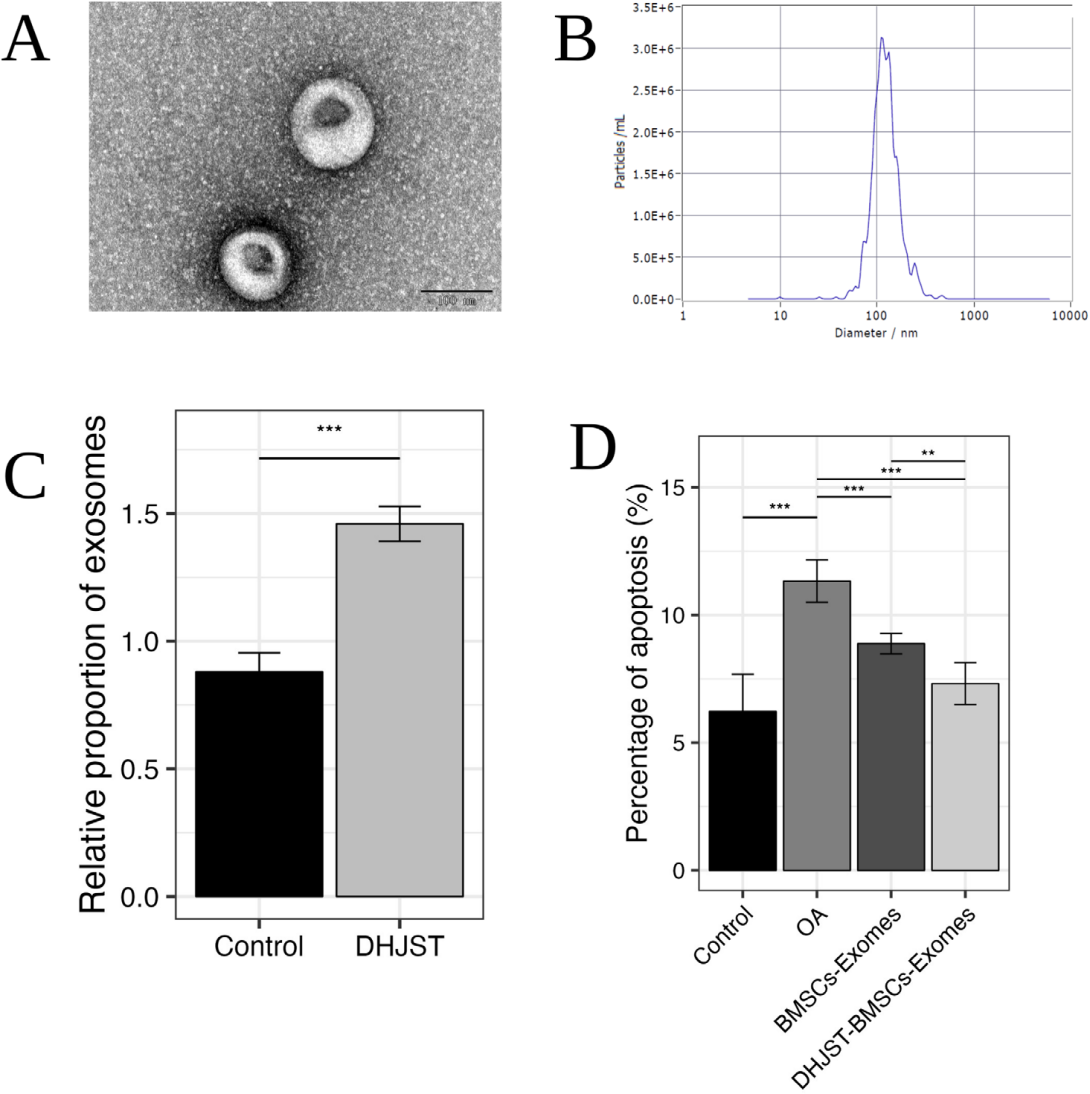
Characterization of DHJSD‐pretreated BMSC exosomes and their anti‐apoptotic effects. (A) Morphological characteristics of DHJSD‐pretreated BMSC‐Exos under transmission electron microscopy (scale bar = 100 nm); (B) Particle size distribution and concentration analysis of exosomes measured by Nanoparticle Tracking Analysis (NTA); (C) Flow cytometry detection of CD9+/CD63+/CD81+ exosome markers; (D) Chondrocyte apoptosis rates measured by flow cytometry across different intervention groups. Data are expressed as mean ± SD (*n* = 3). The difference between the two groups is statistically significant. **P < 0.01, ***P < 0.001.

To investigate the intercellular communication mediated by dietary polyphenols, we first performed a comprehensive characterization of the BMSC‐derived exosomes. TEM observation revealed typical cup‐shaped or ring‐shaped morphology with obvious membrane boundaries (Figure 9A). NTA results further confirmed the purity and size consistency of the samples, with a characteristic peak diameter ranging from 30 to 150 nm, consistent with the standard size distribution of exosomes (Figure 9B). Flow cytometry additionally confirmed the high expression of exosome‐specific markers CD9, CD63, and CD81 (Figure 9C). In the IL‐1β induced OA chondrocyte model, the total apoptosis rate increased significantly in the OA group. However, intervention with DHJST‐Exos markedly reversed this effect, showing superior anti‐apoptotic activity compared to BMSC‐Exos alone (Figure 9D).

**FIGURE 10:**
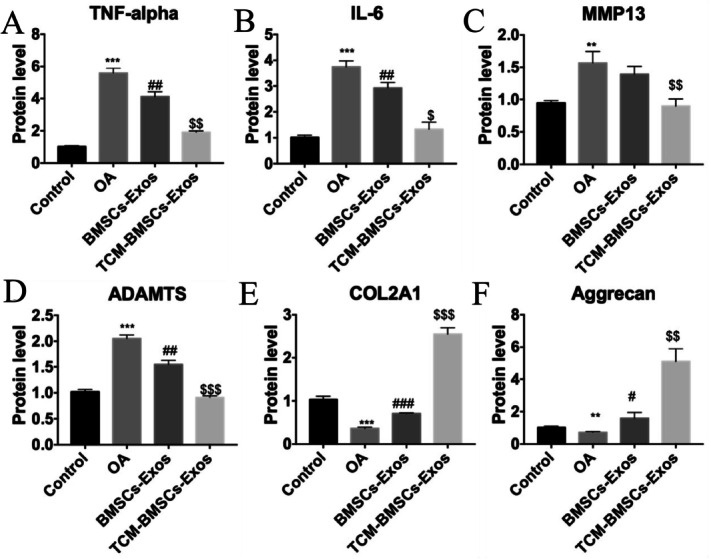
Effects of DHJSD‐Exos on inflammatory cytokines and ECM markers. (A–F) ELISA detection of TNF‐α, IL‐6, MMP‐13, ADAMTS‐5, COL2A1, and Aggrecan. **p < 0.01, ***p < 0.001 versus Control; ^#^ *p* < 0.05, ^##^ *p* < 0.01 versus OA; ^$^ *p* < 0.05, ^$$^ *p* < 0.01 versus BMSCs‐Exos. ^$$$^ *p* < 0.001 versus BMSCs‐Exos.

Furthermore, ELISA results demonstrated that DHJSD‐Exos significantly suppressed the secretion of pro‐inflammatory cytokines, including TNF‐α and IL‐6 (Figure 10A,B). Regarding ECM homeostasis, DHJSD‐Exos treatment led to a significant reduction in matrix‐degrading enzymes (MMP‐13 and ADAMTS‐5) and a concurrent restoration of COL2A1 and Aggrecan levels (Figure 10C–F).

### 3.6 DHJSD‐Exos Activate the KDR‐AKT‐JUN Axis to Regulate Chondroprotective Signaling

**FIGURE 11:**
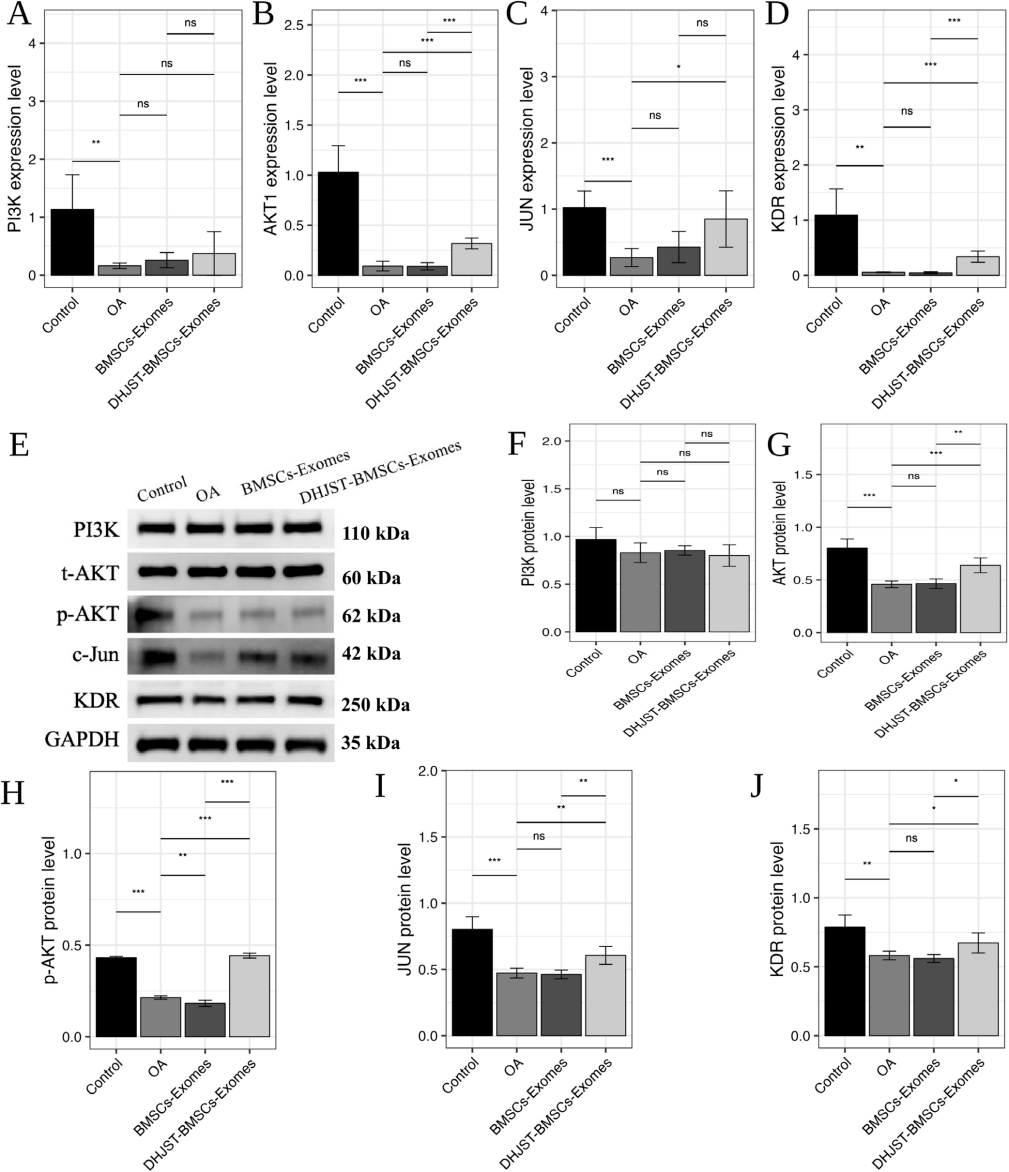
Activation of the KDR‐AKT‐JUN signaling axis by DHJSD‐Exos in OA chondrocytes. (A–D) Quantitative real‐time PCR (RT‐qPCR) analysis showing the mRNA expression levels of *KDR*, *AKT1*, and *JUN* in primary chondrocytes across different treatment groups. (E–J) Representative Western blot images and corresponding quantitative analysis of KDR, JUN, PI3K, and the phosphorylation ratio of AKT (p‐AKT/t‐AKT). GAPDH is used as the internal loading control. Data are presented as mean ± SD (*n* = 3). *p < 0.05, **p < 0.01, ***p < 0.001 versus Control; BMSCs‐Exos.

Based on the initial bioinformatic prediction and molecular docking results (Table 2), we validated the involvement of the KDR‐AKT‐JUN signaling axis. In OA chondrocytes, both mRNA and protein levels of KDR, AKT1, and JUN were significantly down‐regulated (Figure 11A–J). Treatment with DHJSD‐Exos significantly up‐regulated the phosphorylation of AKT (p‐AKT/t‐AKT ratio) and increased the expression of KDR and JUN, whereas the levels of PI3K remained relatively stable.

**FIGURE 12:**
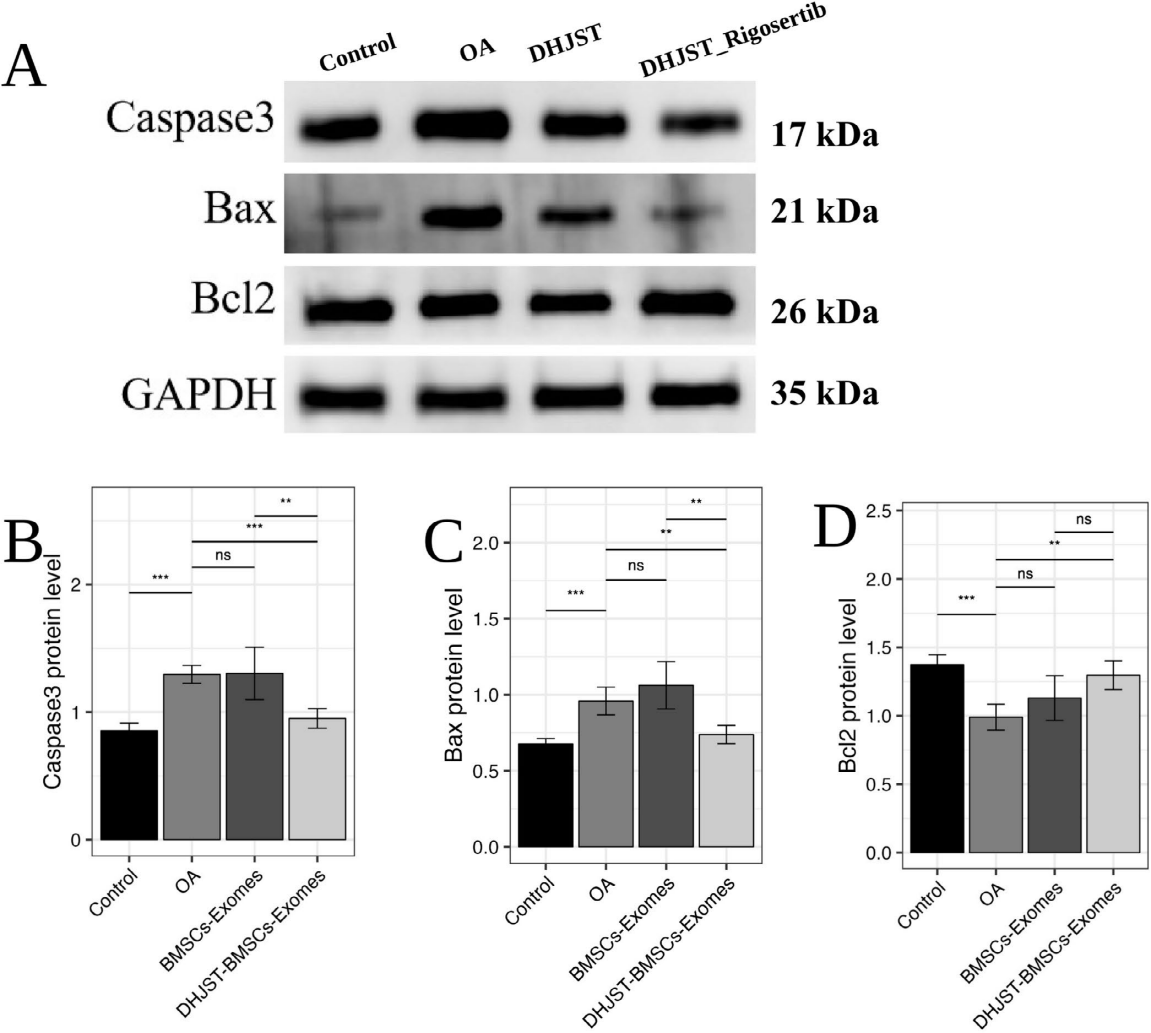
Modulation of downstream apoptotic signaling by DHJST‐Exos. (A) Representative Western blot images of apoptosis‐related proteins: Caspase‐3, Bax, and Bcl‐2 in chondrocytes. (B–D) Densitometric quantification of protein expression levels normalized to GAPDH. DHJSD‐Exos treatment significantly restores the Bcl‐2/Bax ratio, indicating the suppression of mitochondrial apoptosis. Data are expressed as mean ± SD (*n* = 3). Ns *p* > 0.05, *p < 0.05, **p < 0.01, ***p < 0.001.

Furthermore, we examined the downstream apoptotic markers. DHJSD‐Exos intervention significantly down‐regulated the expression of pro‐apoptotic proteins Caspase‐3 and Bax, while up‐regulating the anti‐apoptotic protein Bcl‐2 (Figure 12A–D). These findings confirm that the polyphenolic‐enriched exosomes exert their protective effects by activating the KDR‐AKT‐JUN survival signaling cascade.

### 3.7 In Vivo Validation and Mechanistic Reversal via AKT Inhibition

**FIGURE 13:**
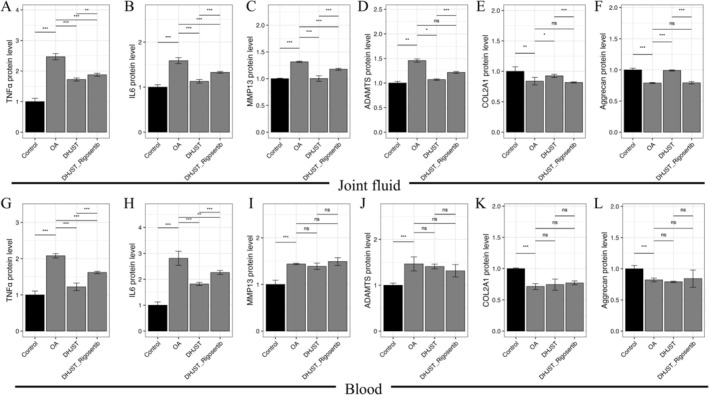
In vivo anti‐inflammatory and matrix‐stabilizing effects of DHJST‐Exos in OA rat models. (A–F) ELISA quantification of pro‐inflammatory cytokines (TNF‐α, IL‐6), matrix‐degrading enzymes (MMP‐13, ADAMTS‐5), and ECM markers (COL2A1, Aggrecan) in the synovial fluid; (G–L) Corresponding protein levels of the aforementioned markers in the peripheral blood. Data are presented as mean ± SD (*n* = 5 per group). *p < 0.05, **p < 0.01 and ***p < 0.001 versus Control.

To validate whether the chondroprotective effects of DHJST‐Exos are predominantly mediated through the AKT signaling axis, we employed a collagenase‐induced OA rat model and the PI3K/AKT‐specific inhibitor, Rigosertib. Intra‐articular administration of DHJST‐Exos significantly suppressed the levels of inflammatory cytokines (TNF‐α and IL‐6) and matrix‐degrading enzymes (MMP‐13 and ADAMTS‐5) in both synovial fluid and peripheral blood, while concurrently preserving the expression of ECM components, COL2A1 and Aggrecan (Figure 13A–L).

**FIGURE 14:**
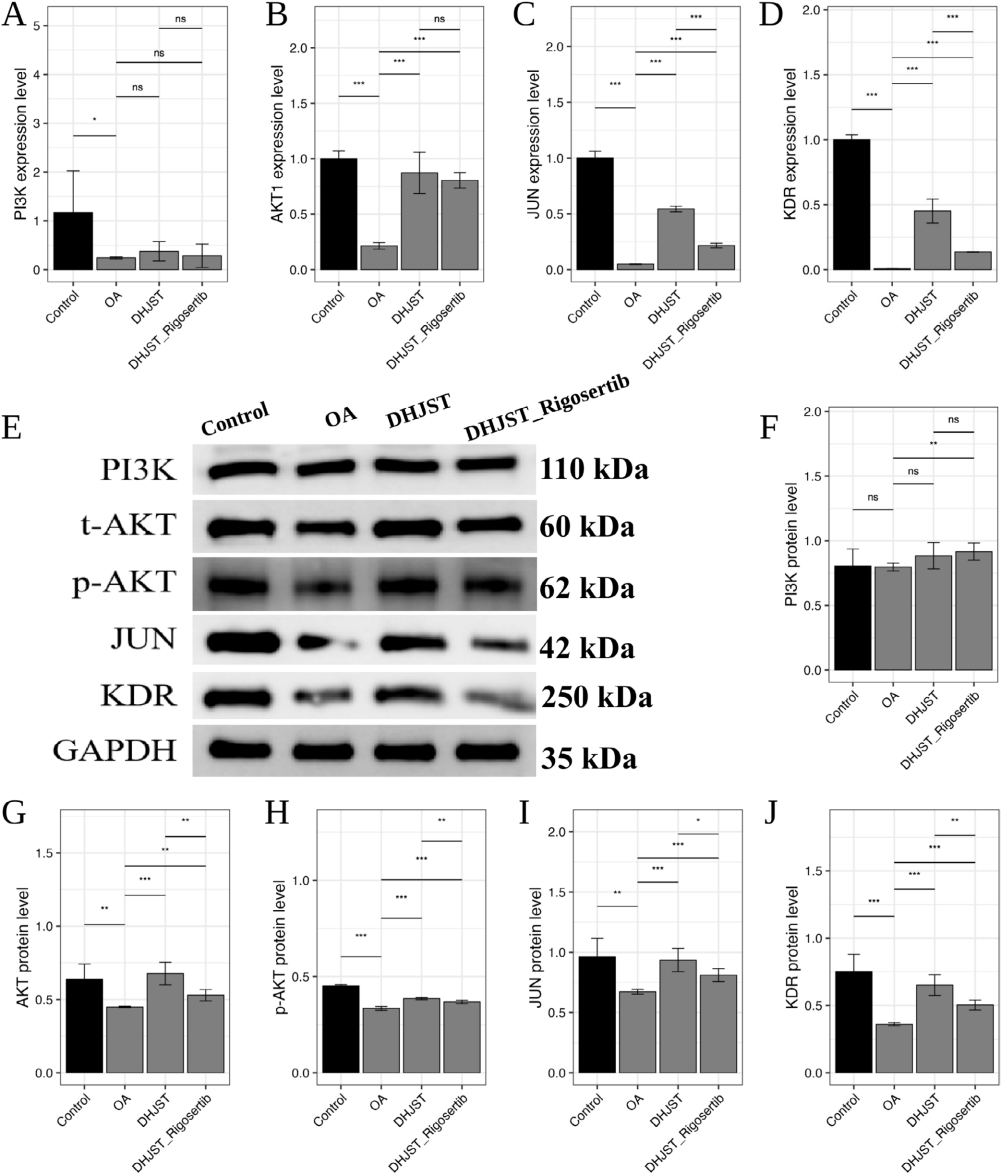
DHJST‐Exos activate the KDR‐AKT‐JUN signaling axis in vivo. (E) Representative Western blot bands showing the expression of KDR, p‐AKT, AKT, and JUN in rat cartilage tissues from different groups. (A–D) Quantitative analysis of KDR/GAPDH, p‐AKT/AKT ratio, and JUN/GAPDH. (E–J) Representative images and quantitative analysis of immunohistochemical (IHC) staining for KDR, p‐AKT, and JUN in the tibial plateau (Scale bar = 100 μm). Data are expressed as mean ± SD (*n* = 3 for Western blot). *p < 0.05, **p < 0.01, ***p < 0.0001 versus Control group.

**FIGURE 15:**
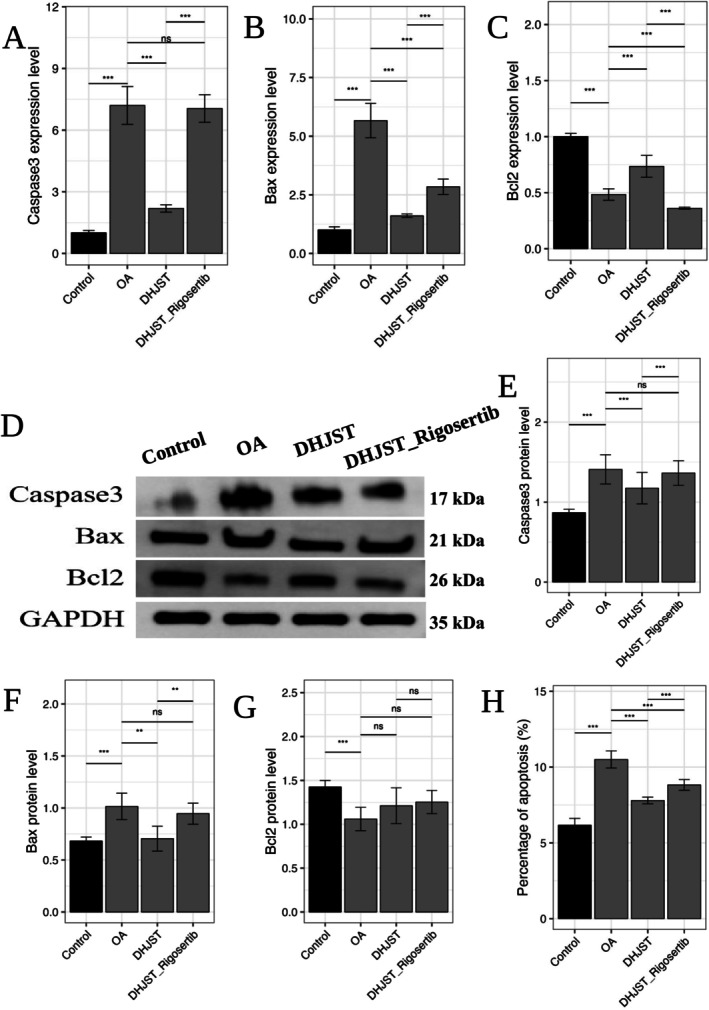
AKT inhibition abrogates the anti‐apoptotic effects of DHJST‐Exos in OA cartilage tissue. (A–C) Transcriptional levels of apoptosis‐related genes *Caspase3*, *Bax*, and *Bcl2* in rat cartilage tissue. (D) Representative Western blot images for Caspase‐3, Bax, and Bcl‐2 proteins. (E–G) Quantitative densitometric analysis of apoptotic protein expression normalized to GAPDH. (H) Changes in the percentage of chondrocyte apoptosis in cartilage tissue across different intervention groups. Compared to the DHJST‐Exos monotherapy group, the Rigosertib combination group exhibited significantly higher levels of pro‐apoptotic markers (Caspase‐3 and Bax) and lower levels of the anti‐apoptotic marker Bcl‐2. These results indicate that the chondroprotective and anti‐apoptotic efficacy of DHJST‐Exos is primarily mediated via AKT signaling. Data are expressed as mean ± SD (*n* = 5). **p < 0.01 and ***p < 0.001 versus Control group.

At the molecular level, DHJST‐Exos treatment robustly enhanced the phosphorylation of AKT and upregulated the expression of JUN and KDR in rat cartilage tissue (Figure 14A–J), consistent with our in vitro findings. Crucially, the therapeutic benefits of DHJST‐Exos, including the attenuation of chondrocyte apoptosis and the maintenance of the Bcl‐2/Bax ratio, were significantly abrogated by the co‐administration of Rigosertib (Figure 15A–H). These results demonstrate that the activation of the KDR–AKT–JUN signaling cascade is a primary mechanism through which DHJST‐Exos exert their chondroprotective efficacy in vivo.

## 4 Discussion

Duhuo Jisheng Decoction (DHJST), a polyphenol‐rich traditional herbal formula, has demonstrated significant application in clinical management of osteoarthritis (OA). Its historical roots trace back to the 7th century AD, as it was initially recorded in “Essential Prescriptions for Emergencies”. Comprising 15 diverse Chinese herbal components—including *Angelicae Pubescentis Radix* (Duhuo), *Taxilli Herba* (Sangjisheng), *Eucommiae Cortex* (Duzhong), *Achyranthes Bidentatae Radix* (Niuxi), and *Asari Radix cum Rhizoma* (Xixin)—this decoction exhibits a range of pharmacological effects, such as immunomodulation, enhancement of liver and kidney functions, and alleviation of pain. In the context of OA, the clinical efficacy of DHJST has been documented to exceed 85% (Zhang et al. 2016; Ahmed et al. 2022). Clinical observations have evidenced that this decoction effectively suppresses pro‐inflammatory cytokines, namely IL‐6 and TNF‐α, while concurrently mitigating joint pain and stiffness resulting from morphological bone changes (Zhang et al. 2016; Ahmed et al. 2022). Additionally, our study revealed a marked decrease in the levels of IL‐6, TNF‐α, and MMP13 in IL‐1β‐induced chondrocytes and OA rat synovial fluid following DHJST intervention. Furthermore, we noted that DHJST‐Exos treatment significantly restored the expression of Aggrecan and COL2A1. Given that the loss of extracellular matrix (ECM) components signifies chondrocyte senescence and OA progression, these findings suggest the potent role of DHJST bioactives in enhancing chondrocyte vitality and maintaining ECM homeostasis. These results align with current pharmacological research and further reinforce the therapeutic potential of DHJST in the management of chronic degenerative joint diseases.

Our primary research centered on elucidating the influence of DHJST on the functional modulation of BMSC‐derived exosomes. BMSCs play a pivotal role in cartilage regeneration following injury, primarily through the paracrine secretion of bioactive factors and extracellular vesicles that target specific effector cells. Previous studies have demonstrated that interventions utilizing BMSC‐derived supernatants or exosomes can mitigate chondrocyte inflammatory responses, impede apoptosis, and promote the expression of ECM components, such as type II collagen (Kim et al. 2020; Cao et al. 2023). In various animal models, these exosomal interventions significantly alleviate pain and inflammation in OA rats, enhance joint structural integrity, and facilitate the healing of subchondral bone and cartilage (Zhang et al. 2019; He et al. 2020). Consistent with these findings, our results showed that BMSC‐Exos treatment led to a significant reduction in pro‐inflammatory cytokine levels and apoptosis rates in IL‐1β‐stimulated chondrocytes. Notably, exosomes derived from BMSCs preconditioned with DHJST‐containing serum (DHJST‐Exos) exhibited a more potent inhibitory effect on chondrocyte inflammation and apoptosis compared to standard BMSC‐Exos. This observation indicates that the bioactive constituents in DHJST may potentially amplify the therapeutic cargo of BMSC‐derived exosomes, thereby enhancing their chondroprotective efficacy in the management of OA.

Our findings suggest that the therapeutic effect of DHJST‐Exos on OA is primarily mediated through PI3K/AKT activation, which subsequently modulates downstream effectors such as JUN—a pivotal transcription factor and a downstream signal of AKT (Zhao et al. 2015). While JUN (as part of the AP‐1 complex) is traditionally associated with pro‐apoptotic signaling in certain OA models (Karin et al. 1997; Wisdom et al. 1999; Raivich 2008; Rossler et al. 2002; Lu et al. 2014), its biological role is highly context‐dependent and regulated by site‐specific phosphorylation (Karin et al. 1997; Wisdom et al. 1999). Specifically, while non‐phosphorylable JUN mutants may trigger apoptosis (Huang et al. 2000), phosphorylation at Ser‐63 and Ser‐73 has been shown to inhibit PTEN and support cell survival, further synergizing with AKT signaling to suppress apoptotic cascades (Hettinger et al. 2007; Potapova et al. 2001).

In the present study, the downregulated expression of JUN in the OA model and its significant restoration following DHJST‐Exos treatment—coupled with the concomitant decrease in Caspase‐3/Bax and the reversal of chondrocyte apoptosis—suggests that in this specific therapeutic context, JUN acts as a protective mediator. This shift towards a pro‐survival role likely underpins the chondroprotective efficacy of the DHJST‐Exos/AKT/JUN axis. However, whether KDR and JUN serve as direct targets of DHJST‐derived polyphenols or are upregulated secondary to AKT activation remains to be further elucidated. The partial reversal of these effects by Rigosertib further reinforces the involvement of the AKT pathway in this protective mechanism.

Furthermore, through the integration of network pharmacology and unsupervised machine learning, we identified KDR as another pivotal protein in OA pathogenesis. KDR encodes the Vascular Endothelial Growth Factor Receptor 2 (VEGFR2), which is expressed across diverse cell types, including endothelial cells, fibroblasts, and chondrocytes. Activated by growth factors such as VEGFA, VEGFC, and VEGFD, KDR plays a crucial role in regulating angiogenesis, inflammatory responses, and cellular survival pathways (Peach et al. 2018). Notably, our study revealed a significant downregulation of KDR expression in OA chondrocytes and cartilage tissue, aligning with findings reported by A. Giatromanolaki et al. (2001) and M. Ikeda et al. (2000). This downregulation is particularly significant as KDR activation triggers the phosphorylation of the AKT signaling pathway (Simons et al. 2016; Ruan and Kazlauskas 2012), ultimately contributing to chondroprotection (Mathews and Berk 2008). These observations suggest that DHJST‐Exos may activate the KDR‐AKT‐JUN signaling cascade, thereby inhibiting chondrocyte apoptosis in OA.

Despite these insights, several limitations of the current study should be acknowledged. First, regarding mechanistic validation, Rigosertib was employed as a pharmacological inhibitor of the PI3K/AKT pathway. While Rigosertib effectively suppressed AKT phosphorylation in our model, it is a multi‐kinase inhibitor that may influence other signaling nodes; thus, we cannot entirely exclude the contributory roles of other kinases. Furthermore, although our bioinformatic screening and molecular docking identified JUN and KDR as key responsive elements with high binding affinities for DHJST‐derived polyphenols like quercetin, wogonin, and kaempferol, the present study did not perform genetic loss‐of‐function (e.g., siRNA knockdown) or gain‐of‐function experiments. Consequently, the indispensable necessity of these targets in the therapeutic action of DHJST‐Exos remains preliminary and warrants further confirmation through gene‐silencing techniques.

Additionally, as the precise concentrations and exosomal enrichment of specific bioactive polyphenols were not directly quantified in this study, their individual and synergistic contributions remain hypothetical. While the regulatory interplay of the KDR‐AKT‐JUN axis is documented in other disease models, the exact molecular mechanisms by which KDR activation modulates downstream JUN specifically within the OA microenvironment remain to be fully elucidated. These areas constitute a crucial focus for our future investigations as we continue to refine this combinatorial strategy for osteoarthritis management.

## 5 Conclusion

In summary, this study demonstrates that exosomes derived from BMSCs pre‐conditioned with Duhuo Jisheng Decoction (DHJST)‐containing serum exhibit enhanced anti‐inflammatory and anti‐apoptotic effects on both osteoarthritic chondrocytes and cartilage tissue. These chondroprotective benefits are closely associated with the synergistic activation of the KDR–AKT–JUN signaling axis and can be significantly attenuated by pharmacological inhibition of the AKT pathway. Our findings suggest that pre‐treating BMSCs with polyphenol‐rich dietary bioactives from DHJST effectively improves the therapeutic potential of their secreted exosomes. This synergistic strategy, combining herbal bioactives with exosome‐mediated delivery, represents a promising adjunctive approach for the clinical management of chronic degenerative joint diseases, pending further validation in large‐scale clinical trials.

## Author Contributions

**Zhouwei Liao:** writing – original draft, writing – review and editing, investigation. **Hui Xie:** project administration, writing – review and editing, writing – original draft. **Lixin Wang:** data curation. **Shaoqun Zhang:** methodology. **Shunwan Jiang:** conceptualization. **Jun Liu:** funding acquisition. **Xinqiang Ni:** formal analysis, project administration. **Tian Yu:** formal analysis, visualization. **Junzheng Yang:** resources. **Weiran Wang:** investigation, writing – original draft, writing – review and editing.

## Funding

The project was supported by the Fund of the National Natural Science Foundation of China (Hui Xie, No. 82104887), Guangdong Basic and Applied Basic Research Foundation (Jun Liu, No. 2022A1515220131), the National Natural Science Foundation of China (Jun Liu, No. 81873314), Project of Administration of Traditional Chinese Medicine of Guangdong Province of China (Tian Yu, No. 20221350), the National Natural Science Foundation of China (Xinqiang Ni, No. 82374517), Basic Research on Shenzhen Science and Technology Innovation Projects (General Projects, Shaoqun Zhang, No. JCYJ20210324111613037).

## Ethics Statement

The project design complies with the ethical requirements of animal experiments at Guangzhou University of Traditional Chinese Medicine. The apply ID is 20210304032.

## Consent

All authors in this study consent to publish this article and the related data.

## Conflicts of Interest

The authors declare no conflicts of interest.

## Supporting information


**Figure S1:** Gene set variation analysis (GSVA) of PI3K/AKT and cell death pathways.
**Figure S2:** Network analysis of the targeting relationships between DHJST bioactive ingredients and OA hub genes.
**Figure S3:** Pearson correlation analysis of DHJST targets and OA‐specific DEGs.
**Figure S4:** Molecular docking of DHJSD bioactives with IKBKB and IL6.

**Table S1:** Detailed composition of Duhuo Jisheng Decoction (DHJST).

**Table S2:** Included osteoarthritis transcriptome sequencing datasets.

## Data Availability

All data will be available on request.
