# Supplementary material for: Polyphenol‐Rich Duhuo Jisheng Decoction Enhances Mesenchymal Stem Cell–Derived Exosome–Mediated Chondroprotection via PI3K/AKT Signaling in Osteoarthritis
Source: Food Sci Nutr. 2026 May 27;14(6):e71867. doi: 10.1002/fsn3.71867 (PMC13239469; doi:10.1002/fsn3.71867)
Supplement: Supplementary file 1 — Figure S1: Gene set variation analysis (GSVA) of PI3K/AKT and cell death pathways. Figure S2: Network analysis of the targeting relationships between DHJST bioactive ingredients and OA hub genes. Figure S3: Pearson correlation analysis of DHJST targets and OA‐specific DEGs. Figure S4: Molecular docking of DHJSD bioactives with IKBKB and IL6. [file FSN3-14-e71867-s001.zip › Supplementary Figure.docx]

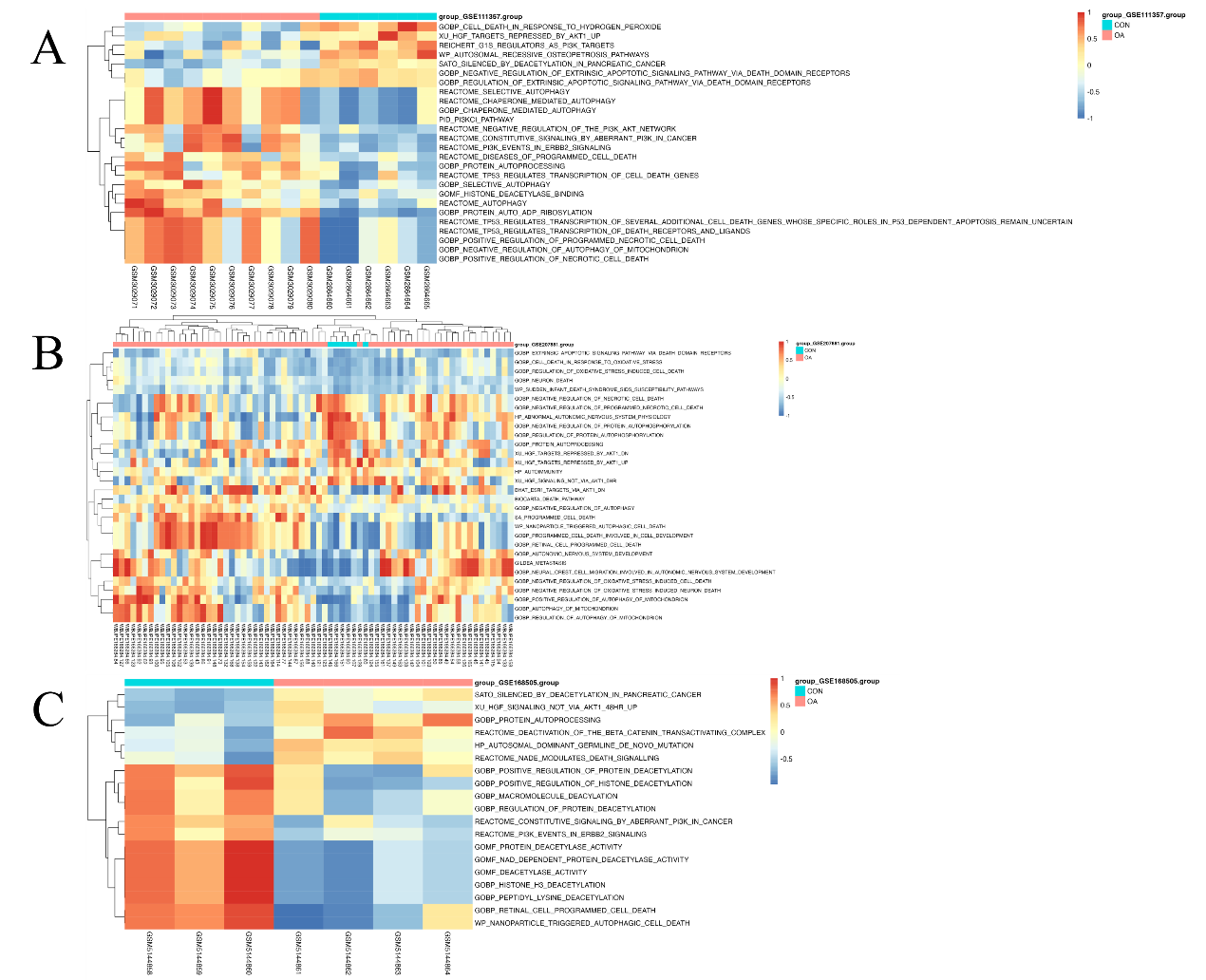


**Supplementary Figure 1. Gene set variation analysis (GSVA) of PI3K/AKT and cell death pathways.** (A–C) Variation scores for AKT and cell death-related gene sets in the GSE111357 (using GSE107308 as control), GSE207881, and GSE168505 datasets. Only gene sets with *adj.P < 0.05* are displayed.


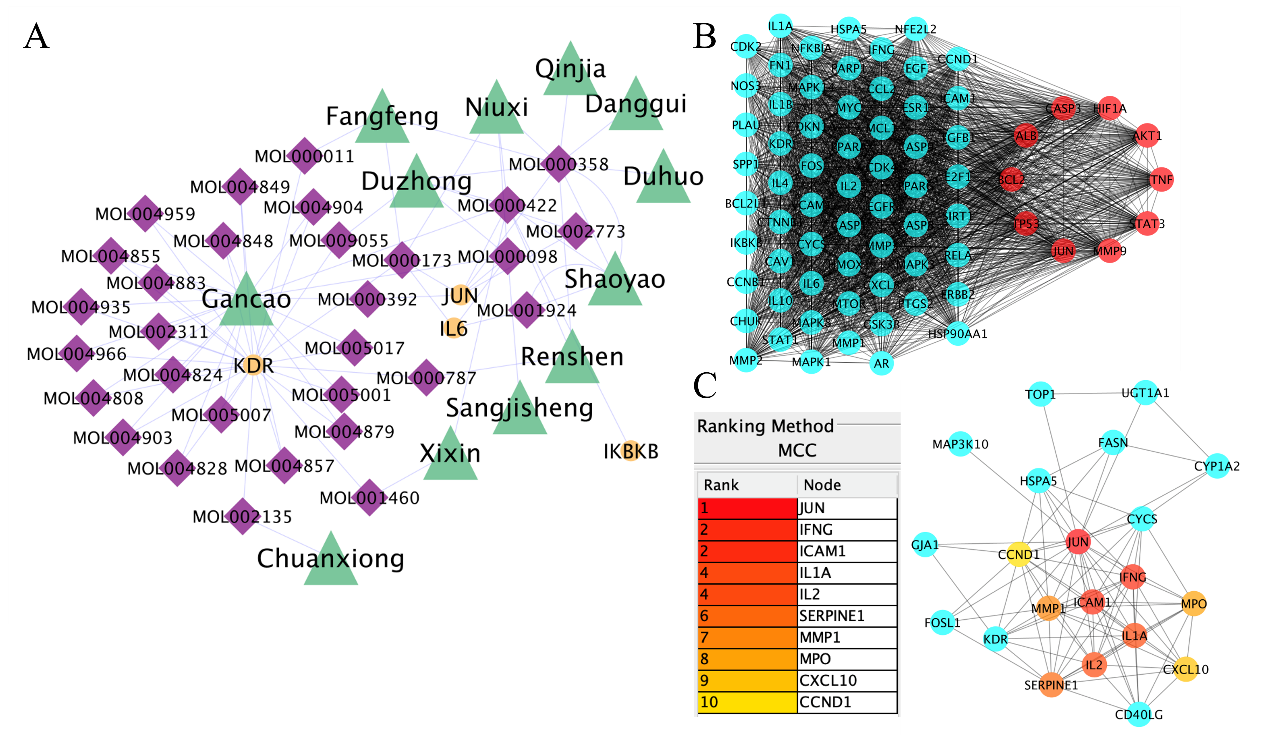


**Supplementary Figure 2. Network Analysis of the Targeting Relationships Between DHJST Bioactive Ingredients and OA Hub Genes.** (A) DHJSD Ingredient–Target Interaction Network. A comprehensive analysis reveals that 26 active ingredients within **DHJST** target four central hub genes: *KDR, IL6, JUN,* and *IKBKB*. Specifically, 21 ingredients are predicted to target *KDR*, 3 target *IL6*, and 4 target *JUN*. Notably, the dietary polyphenol **kaempferol** (MOL000422) exhibited multi-target potential by interacting with both *JUN* and *IKBKB*. In the network visualization, purple squares represent small molecules (identified by TCMSP IDs), green triangles represent the 15 constituent herbs, and yellow circles denote the four core gene nodes. (B) Identification of Core Target Clusters. The primary network cluster of **DHJSD** active ingredient targets is determined through the Maximal Clique Centrality (MCC) algorithm within the Cytohubba plug-in, highlighting the functional importance of these nodes within the overall drug-target interactome. (C) Hub Protein Interaction Sub-network. This panel further delineates the protein-protein interaction (PPI) landscape surrounding **JUN and KDR**, illustrating their respective interacting partners and reinforcing their roles as pivotal signaling mediators in the therapeutic mechanism of DHJST.


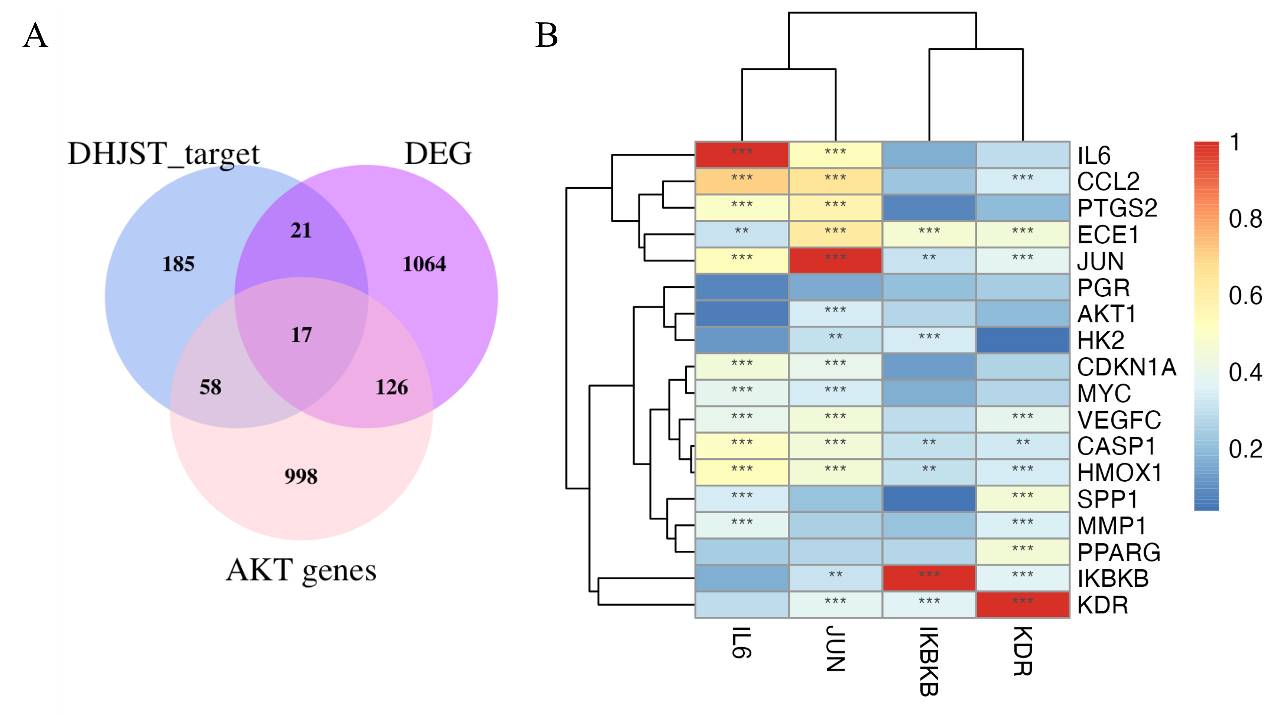


**Supplementary Figure 3. Pearson Correlation Analysis of DHJST Targets and OA-Specific DEGs.**(A) **Overlap Analysis of Functional Gene Sets.** The Venn diagram illustrates the degree of overlap among **Duhuo Jisheng Decoction (DHJST)** targets, AKT signaling-related genes, and DEGs identified in osteoarthritis (OA) cartilage tissue. A total of 75 intersections are identified between DHJSD targets and AKT signaling genes, 17 of which exhibit significant differential expression in OA. (B) **Correlation Matrix of Core Genes.** This panel highlights the expression patterns of four core hub genes: *KDR, IL6, IKBKB,* and *JUN*. The Pearson correlation analysis reveals a moderate positive correlation between the core gene *JUN* and *AKT1*. Statistical significance levels are indicated by asterisks: **P < 0.01 and ***P < 0.001.


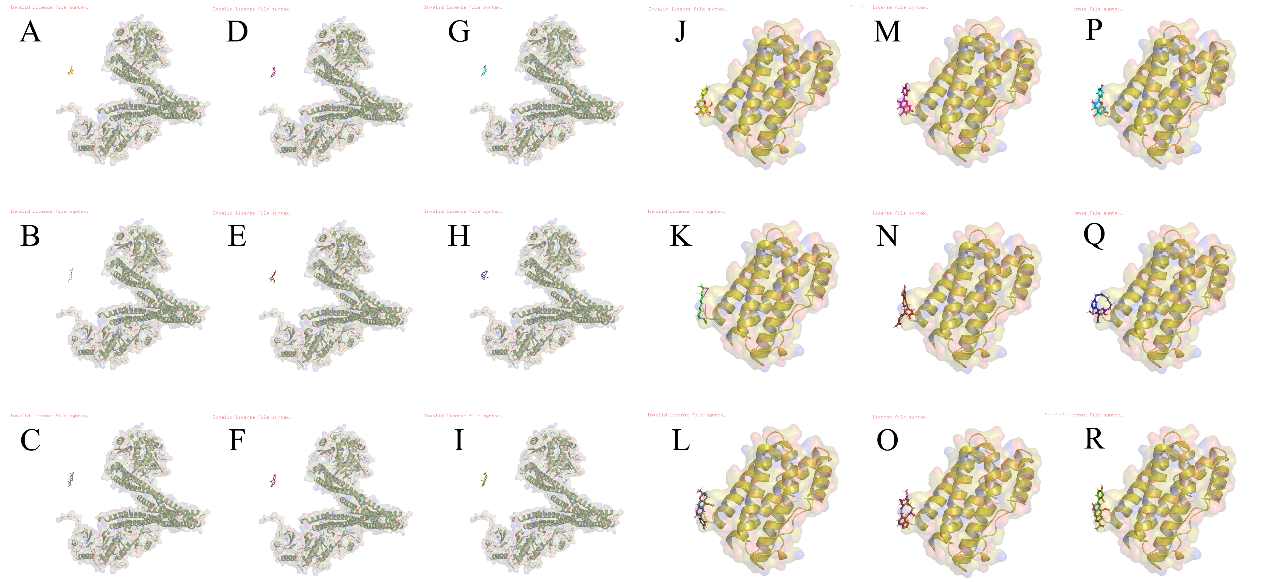


**Supplementary Figure 4. Molecular Docking of DHJSD Bioactives with IKBKB and IL6.**

Molecular docking simulations reveal that representative small molecules from DHJSD exhibit relatively weak binding affinities for IKBKB and IL6, suggesting these are not the primary direct targets of the formula.

(A–I) **Docking Profiles with IKBKB.** Schematic diagrams of molecular docking results between **IKBKB** and nine bioactive constituents: wogonin (A), quercetin (B), kaempferol (C), hirsutin_qt (D), Sigmoidin-B (E), Myricanone (F), licopyranocoumarin (G), Cryptopin (H), and (2S)-6-(2,4-dihydroxyphenyl)-2-(2-hydroxypropan-2-yl)-4-methoxy-2,3-dihydrofuro[3,2-g]chromen-7-one (I).

(J–R) **Docking Profiles with IL6.** Schematic diagrams of the interactions between **IL6** and the aforementioned nine dietary small molecules. The calculated binding energies for these interactions are generally higher (weaker affinity) compared to those observed for KDR and JUN.
