# Supplementary figures and images for: Polyphenol‐Rich Duhuo Jisheng Decoction Enhances Mesenchymal Stem Cell–Derived Exosome–Mediated Chondroprotection via PI3K/AKT Signaling in Osteoarthritis
Source: Food Sci Nutr. 2026 May 27;14(6):e71867. doi: 10.1002/fsn3.71867 (PMC13239469; doi:10.1002/fsn3.71867)

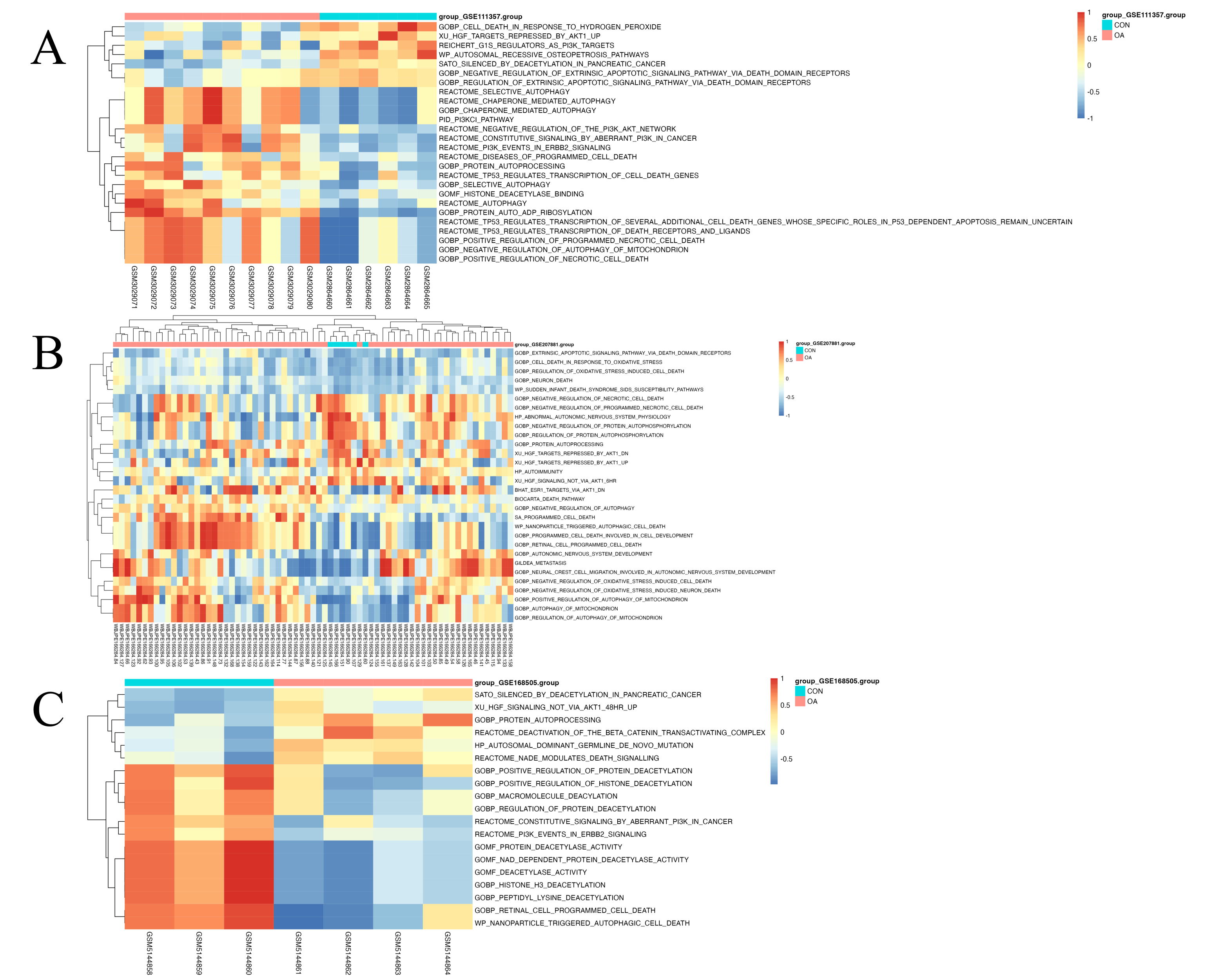

Supplement: Supplementary file 1 — Figure S1: Gene set variation analysis (GSVA) of PI3K/AKT and cell death pathways. Figure S2: Network analysis of the targeting relationships between DHJST bioactive ingredients and OA hub genes. Figure S3: Pearson correlation analysis of DHJST targets and OA‐specific DEGs. Figure S4: Molecular docking of DHJSD bioactives with IKBKB and IL6. [file FSN3-14-e71867-s001.zip › fsn371867-sup-0001-Supinfo1@S1.png]

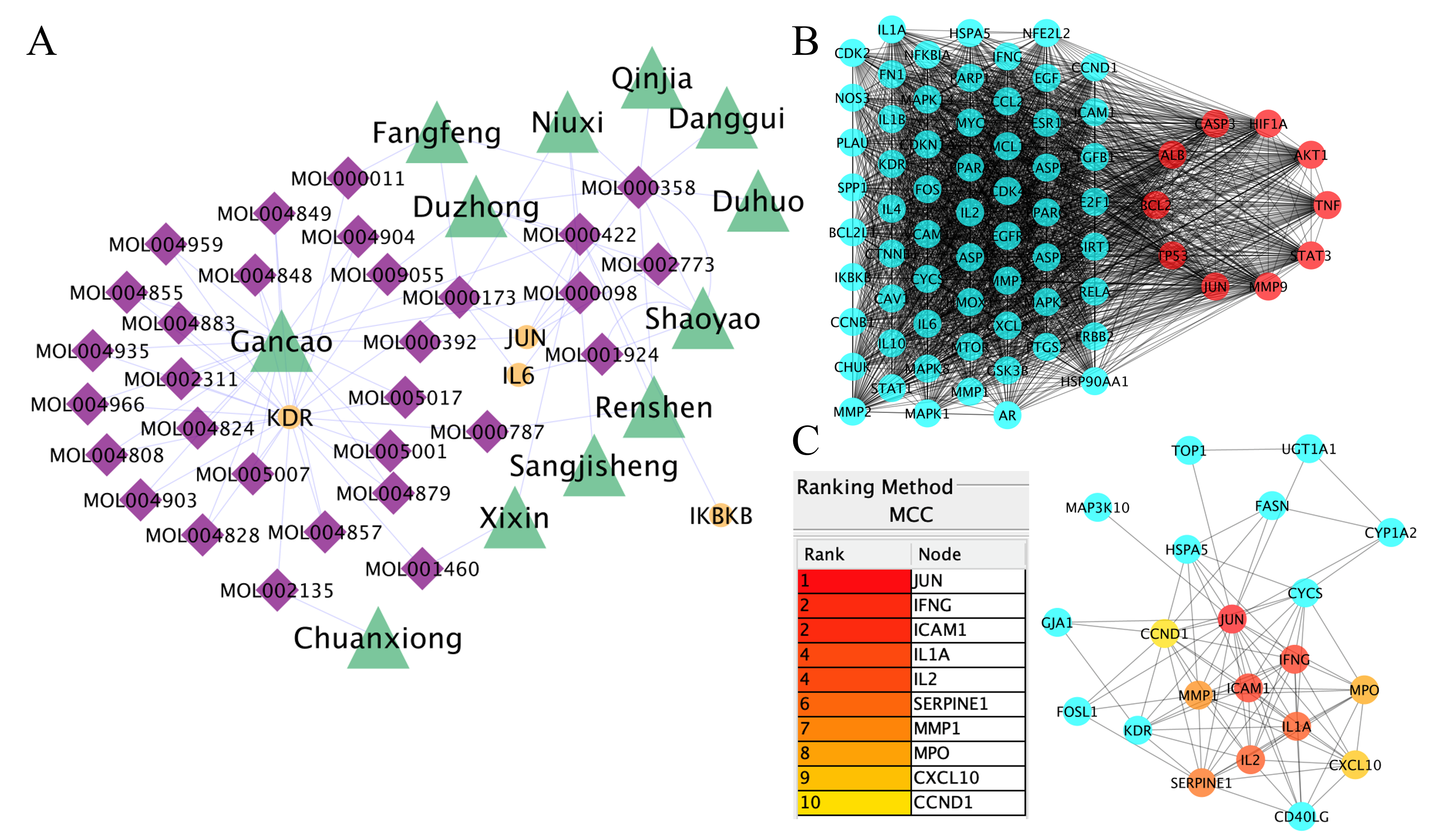

Supplement: Supplementary file 1 — Figure S1: Gene set variation analysis (GSVA) of PI3K/AKT and cell death pathways. Figure S2: Network analysis of the targeting relationships between DHJST bioactive ingredients and OA hub genes. Figure S3: Pearson correlation analysis of DHJST targets and OA‐specific DEGs. Figure S4: Molecular docking of DHJSD bioactives with IKBKB and IL6. [file FSN3-14-e71867-s001.zip › fsn371867-sup-0002-Supinfo2@S2.png]

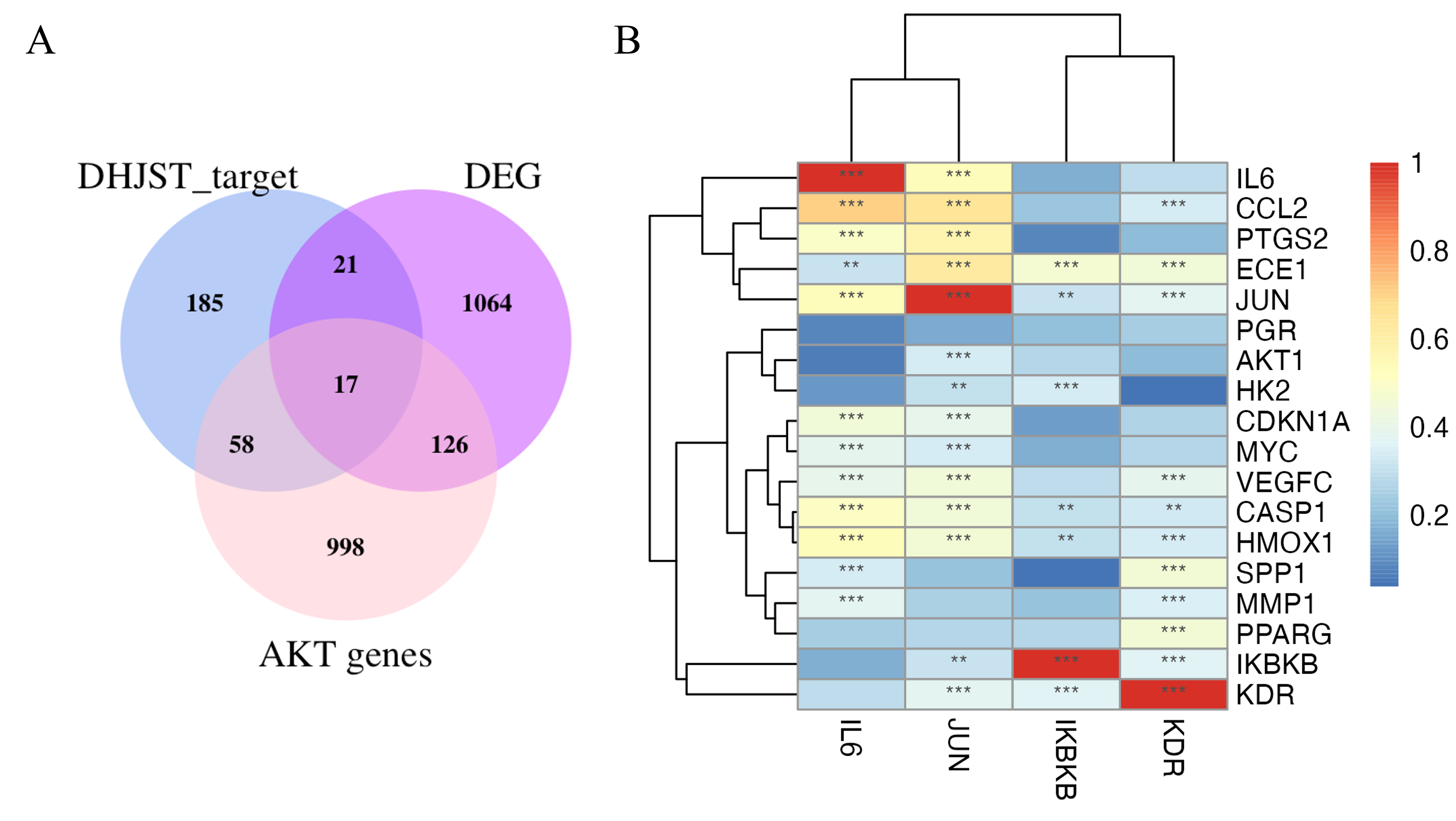

Supplement: Supplementary file 1 — Figure S1: Gene set variation analysis (GSVA) of PI3K/AKT and cell death pathways. Figure S2: Network analysis of the targeting relationships between DHJST bioactive ingredients and OA hub genes. Figure S3: Pearson correlation analysis of DHJST targets and OA‐specific DEGs. Figure S4: Molecular docking of DHJSD bioactives with IKBKB and IL6. [file FSN3-14-e71867-s001.zip › fsn371867-sup-0003-Supinfo3@S3.png]

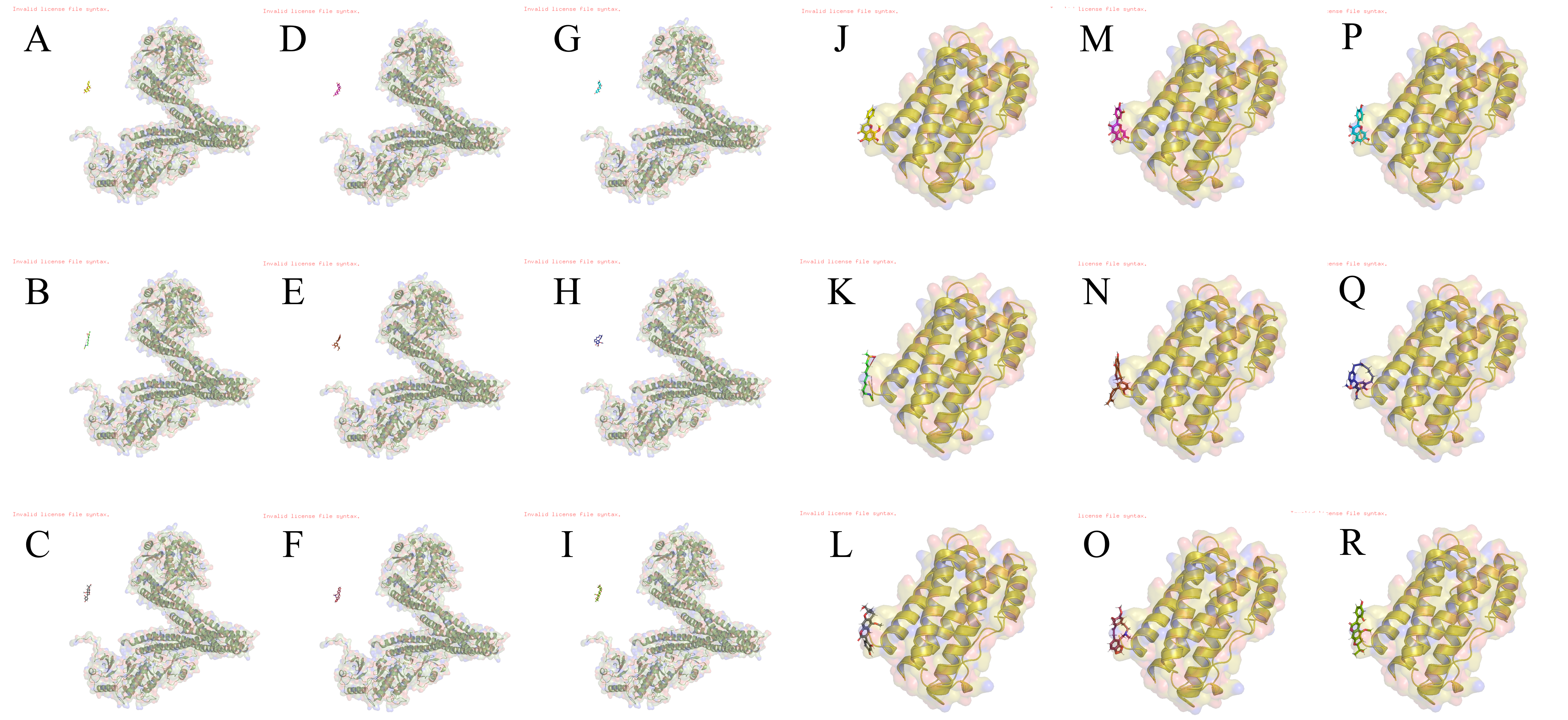

Supplement: Supplementary file 1 — Figure S1: Gene set variation analysis (GSVA) of PI3K/AKT and cell death pathways. Figure S2: Network analysis of the targeting relationships between DHJST bioactive ingredients and OA hub genes. Figure S3: Pearson correlation analysis of DHJST targets and OA‐specific DEGs. Figure S4: Molecular docking of DHJSD bioactives with IKBKB and IL6. [file FSN3-14-e71867-s001.zip › fsn371867-sup-0004-Supinfo4@S4.png]
